# Enhanced ASGR2 by microplastic exposure leads to resistance to therapy in gastric cancer

**DOI:** 10.7150/thno.73226

**Published:** 2022-04-04

**Authors:** Hyeongi Kim, Javeria Zaheer, Eui-Ju Choi, Jin Su Kim

**Affiliations:** 1 Division of RI Application, Korea Institute Radiological and Medical Sciences, Seoul 01812, Republic of Korea.; 2 Department of Life Sciences, School of Life Sciences and Biotechnology, Korea University, Seoul 02841, Republic of Korea.; 3 Radiological and Medico-Oncological Sciences, University of Science and Technology (UST), Seoul 01812, Republic of Korea.

**Keywords:** Microplastics, gastric cancer, cancer hallmarks, polystyrene, ASGR2

## Abstract

**Background:** Microplastics (MPs) are a new global environmental threat. Previously, we showed the biodistribution of MPs using [^64^Cu] polystyrene (PS) and PET in mice. Here, we aimed to identify whether PS exposure has malignant effects on the stomach and induces resistance to therapy.

**Methods:** BALB/c nude mice were fed 1.72 × 10^4^ particles/mL of MP. We investigated PS accumulation in the stomach using radioisotope-labeled and fluorescent-conjugated PS. Further, we evaluated whether PS exposure induced cancer stemness and multidrug resistance, and whether it affected tumor development, tumor growth, and survival rate *in vivo* using a 4-week PS-exposed NCI-N87 mouse model. Using RNA-Seq analysis, we analyzed whether PS exposure induced gene expression changes in gastric tissues of mice.

**Results:** PET imaging results showed that a single dose of [^64^Cu]-PS remained for 24 h in the mice stomach. The 4-week daily repetitive dose of fluorescent conjugated PS was deposited in the gastric tissues of mice. When PS was exposed, a 2.9-fold increase in migration rate was observed for NCI-N87 cells. Immunocytochemistry results showed decreased E-cadherin and increased N-cadherin expression, and flow cytometry, qPCR, and western blot analysis indicated a 1.9-fold increase in N-cadherin expression after PS exposure**.** Further, PS-induced multidrug resistance to bortezomib, paclitaxel, gefitinib, lapatinib, and trastuzumab was observed in the NCI-N87 mouse model due to upregulated CD44 expression. RNA-seq results identified increased asialoglycoprotein receptor 2 (*ASGR2*) expression after PS exposure, and *ASGR2* knockdown decreased cell proliferation, migration, invasion, and drug resistance.

**Conclusion:** We demonstrated that *ASGR2* enhanced cancer hallmarks on PS exposure and induced resistance to chemo- and monoclonal antibody-therapy. Our preclinical findings may provide an incentive for further epidemiological studies on the role of MP exposure and its association with gastric cancer.

## Introduction

Microplastics (MPs) with a diameter less than 5 mm are recognized as a new environmental threat and human health risk 1. An analysis of tap water samples from around the world found that a high proportion of drinking water is contaminated with MPs (83% of samples collected worldwide, up to 94% in the USA) 2. MP contamination of food can no longer be ignored 3, 4. MP in food and contamination of MP and during food processing and cooking was reported 5, 6. MP are a ubiquitous global contaminant, identified throughout the marine environment, including seawater, sediment and biota 7. Pre/post-natal exposure to MP as a potential risk factor for autism spectrum disorder was reported 8.

A potential route of MP exposure in the human body is inhalation 9. A previous study showed that the inhalation of synthetic fibers or silk increased the relative risk of cancer of the digestive system by 1.46-fold 10 and that textile dust exposure elevated gastric cancer risk 11. Another possible route of MP exposure in humans is ingestion 9. Based on foodstuffs consumption, the estimated intake of MP is 39,000-52,000 particles person^-1^ year^-1^ 12. MPs were detected in human stool samples 13 and human colectomy specimens 14, providing direct evidence of ingestion in the human diet. The stomach would be the primary exposure site when MPs are ingested 15. Recently, we first identified the absorption path and distribution of MPs using positron emission tomography. Polystyrene was labeled with Copper-64 ([^64^Cu], to yield [^64^Cu]Cu-DOTA-polystyrene) and then orally administered to mice. A single dose of [^64^Cu]Cu-DOTA-polystyrene remained for up to 24 h in the stomach 16.

Gastric cancer is one of the leading causes of cancer-related death worldwide 17. Although the overall survival of most gastric cancer patients is continuously enhanced, metastatic gastric cancer outcomes are poor, with a median survival of approximately 1 year 17. MPs can interact with the stomach and pose a potential hazard as regards food web transmission 16. Although numerous studies have investigated the effects of PS, it is unclear what effect they have on the stomach.

Thus, we hypothesized that MP exposure increases the potential risks of gastric cancer. After exposure of MP polystyrene, the expression level of asialoglycoprotein receptor 2 (ASGR2) gene was enhanced. Then enhanced ASGR2 induced typical cancer hallmarks such as proliferation, N-cadherin, CD44, PD-L1 for both *in vitro* and *in vivo*. After exposure to MP polystyrene, decreased survival rate and increased tumor growth were also observed. To our best knowledge, it is the first study to reveal potential risk of MP to stomach.

## Results

### PS accumulation in the stomach

**Figure 1:**
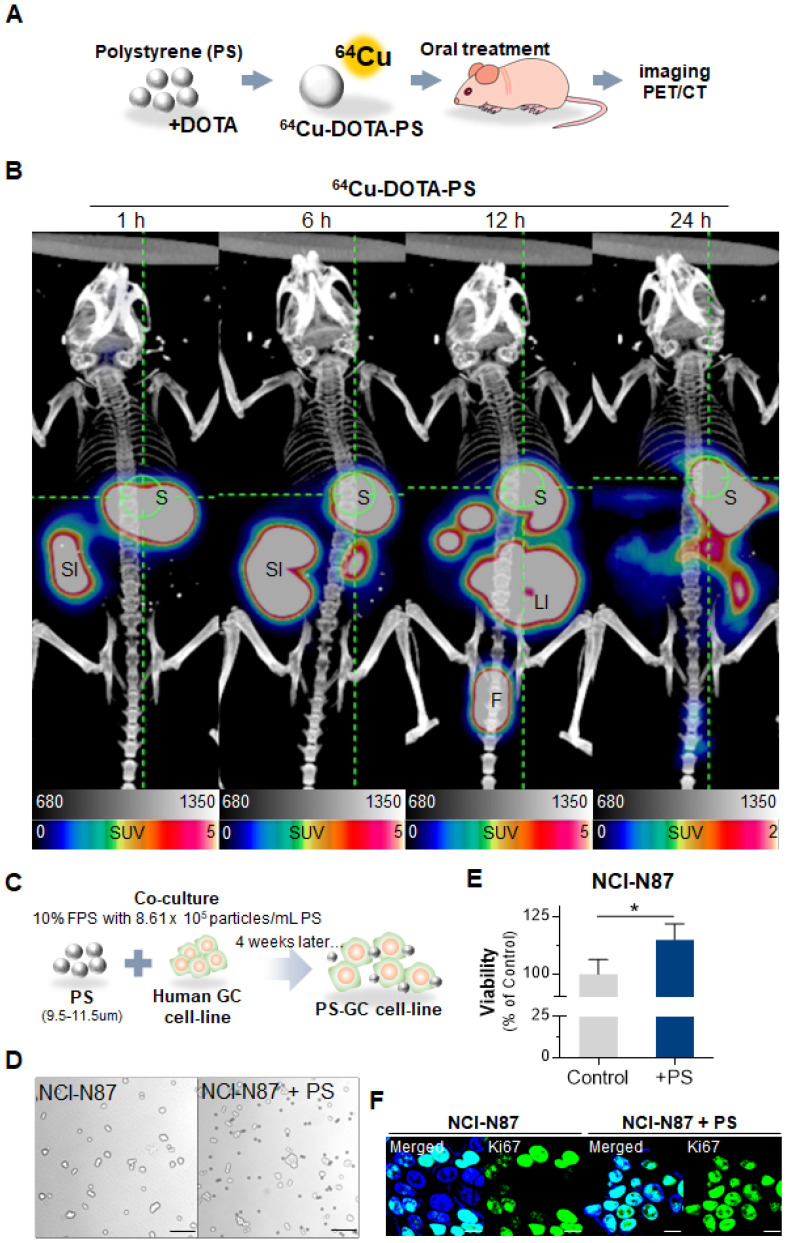
**Polystyrene accumulated in mouse gastric tissue and promoted expression changes. (A-B)** PET Imaging of polystyrene (PS) accumulation in the stomach using [^64^Cu]Cu-DOTA-PS. [^64^Cu]Cu-DOTA-PS (4.81 MBq/57.8 µg/100 µL) was orally administered to mice. PET/CT scans were then acquired at 1 h, 6 h, 12 h, and 24 h after [^64^Cu]Cu-DOTA-PS administration. **(C)** Schematic of PS exposure in human gastric cancer cell lines. PS (8.61 × 10^5^ particles/mL); 10 µm in diameter) was mixed with normal cell culture media (10% FBS) and subcultured for 4 weeks. **(D-E)** Microscopy images of NCI-N87 cells with/without PS exposure and proliferation. PS exposure induced increased proliferation in NCI-N87 cells compared with that in the controls (mean ± standard deviation [SD], * P < 0.05, Student's t-test, Magnification, 20×; scale bar, 100 µm). **(F)** Immunocytochemistry images showing NCI-N87 cells stained for Ki67. PS exposure (10 µm diameter, 8.61 × 10^5^ particles/mL, 4 weeks) increased Ki67 expression in NCI-N87 cells (magnification, 40×; scale bar, 10 µm).

First, we identified the accumulation of polystyrene (PS) in stomach using radioisotope labeled PS and fluorescent conjugated PS. Representative PET images demonstrated that single dose PS, [^64^Cu]Cu-DOTA-polystyrene (4.81 MBq/57.8 μg/100 μL) remained for up to 24 h in the stomach (**Fig. 1A-B**) 16. BALB/c nude mice (5-week-old, male; n = 3) were fed 1.72 × 10^4^ particles/mL of MP, fluorescent green-labeled PS particles, daily for 4 weeks. We then identified PS was accumulated in the gastric tissue of these mice (**Fig. S1A**).

### PS exposure accelerated proliferation, cell invasion and migration

We next investigated whether PS exposure accelerated proliferation in human gastric cancer. Five different human gastric cancer cell lines (AGS, MKN1, MKN45, NCI-N87, and KATOIII) exposed to PS (8.61 × 10^5^ particles/mL) for 4 weeks (**Fig. 1C**). Morphologies of the cells were shown after the 4-week exposure (**Fig. 1D** and **Fig. S1B**). We observed an increase in proliferation in all cell lines (* P < 0.05,** Fig. 1E-F** and **Fig. S1C**).

**Figure 2:**
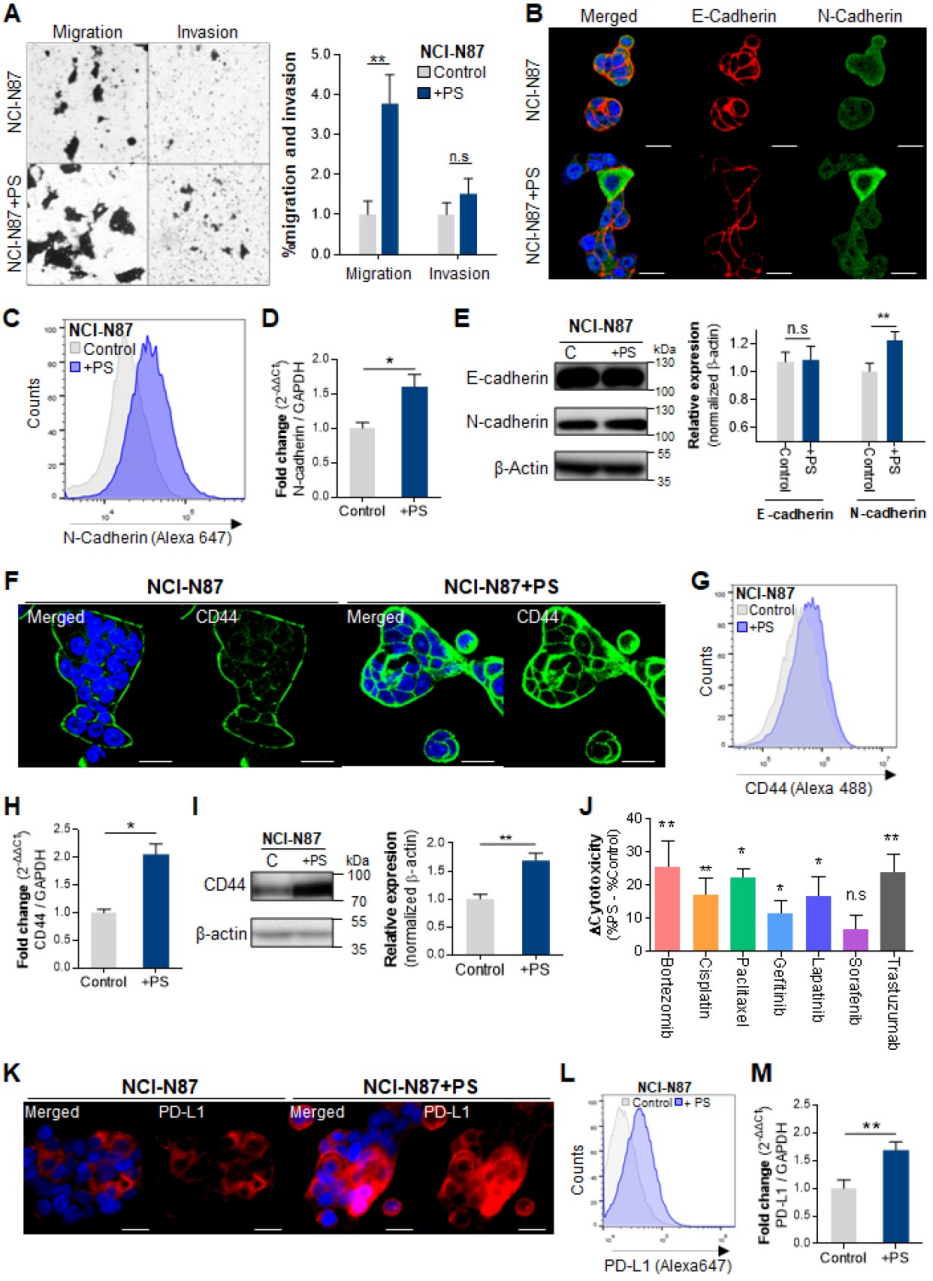
**Polystyrene exposure increased cancer hallmarks. (A)** *In vitro* migration and invasion assays. Bar graphs represent the average number of cells on the underside of the membrane, normalized to the control condition. Polystyrene (PS) promoted the migration of NCI-N87 cells (Magnification, 20×; mean ± SD; * P < 0.05, ** P < 0.005, n.s., not significant, Student's t-test). **(B)** Immunocytochemistry images showing NCI-N87 cells stained for E-cadherin and N-cadherin. PS exposure (10 µm diameter, 8.61 × 10^5^ particles/mL, daily 4 weeks) increased N-cadherin levels in NCI-N87 cells (Magnification, 40×; Scale bar, 20 µm). **(C)** Flow cytometry histograms of N-cadherin expression in gastric cancer cell lines with/without PS exposure. PS exposure upregulated N-cadherin expression in NCI-N87 cells after exposure to PS. **(D)** Quantitative polymerase chain reaction (qPCR) analysis of N-cadherin mRNA expression. N-cadherin mRNA expression increased after PS exposure (mean ± SD, * P < 0.05, Student's t-test). **(E)** Western blot analysis of N-cadherin and E-cadherin expression with/without PS (mean ± SD, ** P < 0.005, n.s., not significant, Student's t-test). **(F)** Immunocytochemistry staining showing CD44 expression in gastric cancer cells (magnification, 40×; scale bar, 20 µm). **(G)** Flow cytometry analysis of CD44 expression in gastric cancer cell lines. Increased CD44 expression for 4 weeks of PS exposure. **(H)** Quantitative polymerase chain reaction (qPCR) analysis of CD44 mRNA expression (mean ± SD, *P < 0.05, Student's t-test). **(I)** Western blot analysis of CD44 expression in cells with/without PS (mean ± SD, ** P < 0.005, n.s., not significant, Student's t-test). **(J)** CD44-induced drug resistance after PS exposure (10 µm diameter, 8.61 × 10^5^ particles/mL, 4 weeks). The cytotoxicity of bortezomib, cisplatin, paclitaxel, Gefitinib, Lapatinib, Sorafenib, or trastuzumab were evaluated. “Δcytotoxicity = %cytotoxicity w/ PS - % cytotoxicity w/o PS” Δcytotoxicity increased for NCI-N87 cell lines with various anti-tumor drugs (* P < 0.05, ** P < 0.005, n.s., not significant. Student's t-test). **(K)** Immunocytochemistry staining of PD-L1 in gastric cancer cells with/without PS (Magnification, 40×; scale bar, 20 µm). **(L)** Flow cytometry histograms of PD-L1 expression in gastric cancer cell lines. PS exposure for 4 weeks dramatically increased PD-L1 expression. **(M)** qPCR analysis of PD-L1 mRNA expression. (mean ± SD, **P < 0.005, Student's t-test).

We investigated whether PS exposure induced invasion and migration *in vitro*. A 2.9-fold of increased migration rate was observed in NCI-N87 cells (** *P* < 0.005, **Fig. 2A**) and in the other cell lines including AGS, MKN1, MKN45, and KATOIII (* *P* < 0.05, ** *P* < 0.005, **Fig. S2A**). An increased invasion rate was also observed in the most cell lines except NCI-N87 (* *P* < 0.05, ** *P* < 0.005, **Fig. 2A** and **Fig. S2A**).

The immunocytochemistry (ICC) results show decreased E-cadherin and increased N-cadherin following PS exposure (**Fig. 2B** and **Fig S2B)**. Flow cytometry demonstrated a 1.9-fold increase in N-cadherin (**Fig. 2C**). The qPCR demonstrated a 1.6-fold increase in N-cadherin gene expression after PS exposure (**Fig. 2D**). The ICC and qPCR analysis results showed that there was no dose effect between 1.72 × 10^5^ and 8.61 × 10^5^ particles/mL on both E-cadherin and N-cadherin expression (**Fig. S4A-C**). The increased N-cadherin expression after PS exposure were also observed in every cell lines (**Fig. S2C-D**)**.** Western blot showed a 1.23-fold increase in N-cadherin after PS exposure (**Fig. 2E**). Similar patterns were observed in other cell lines (**Fig. S2E**)**.**

### PS exposure increased multidrug resistance

CD44+ was identified as cancer stem cells in human gastric cancer 18, 19. We investigated whether PS exposure induced cancer stemness and multidrug resistance. Upregulated CD44 expression was observed using ICC in every cell-line (**Fig. 2F** and **Fig. S3A**). The flow cytometry showed an increase of CD44 in every cell-line (**Fig. 2G** and **Fig. S3B**). Increased mRNA expression and protein expression of CD44 were observed (**Fig. 2H-I** and **Fig. S3C-D**). The ICC and qPCR analysis results showed that there was no dose effect between 1.72 × 10^5^ and 8.61 × 10^5^ particles/mL on CD44 expression (**Fig. S4D-F**).

To assess drug resistance due to CD44, cell cytotoxicity was evaluated using widely used anticancer drugs such as bortezomib, cisplatin, paclitaxel, gefitinib, lapatinib, sorafenib, or trastuzumab. The changes of cytotoxicity were calculated as “Δcytotoxicity = %cytotoxicity w/ PS - % cytotoxicity w/o PS”. As shown in **Fig. 2J**, an increase in Δcytotoxicity (* P < 0.05, ** *P* < 0.005) was apparent, demonstrating PS induced multidrug resistance to bortezomib, cisplatin, paclitaxel, gefitinib, lapatinib, and trastuzumab in NCI-N87. After PS exposure, increased drug resistance was observed based on the IC_50_ assessments (**Fig. S5A**). We found that the IC_50_ value was dramatically increased (**Fig. S5A**), which suggested that the PS exposure induced drug resistance. Multidrug resistance was also shown in AGS, MKN1, MKN45, and KATOIII cell lines after PS exposure (**Fig. S5B**).

In tumor, upregulation of programmed death-ligand 1 (PD-L1, also known as CD274) mediates potent immunosuppressive effects 20, 21. We investigated whether PS exposure increased PD-L1 expression. Upregulated PD-L1 expression was observed using ICC in every cell-line (**Fig. 2K** and **Fig. S6A**). The flow cytometry experiments also showed an increase of PD-L1 in every cell-line (**Fig. 2L** and **Fig. S6B**). PD-L1 mRNA expression was increased (**Fig. 2M** and **Fig. S6C**). Overall, a 4.23-fold increase was observed among the five gastric cancer cell types (**Fig. S6C**).

These results demonstrate that PS exposure increased multidrug resistance and may decrease the response to chemotherapy in patients with gastric cancer.

### PS exposure reinforced cancer hallmarks in *in vivo* mouse model

**Figure 3:**
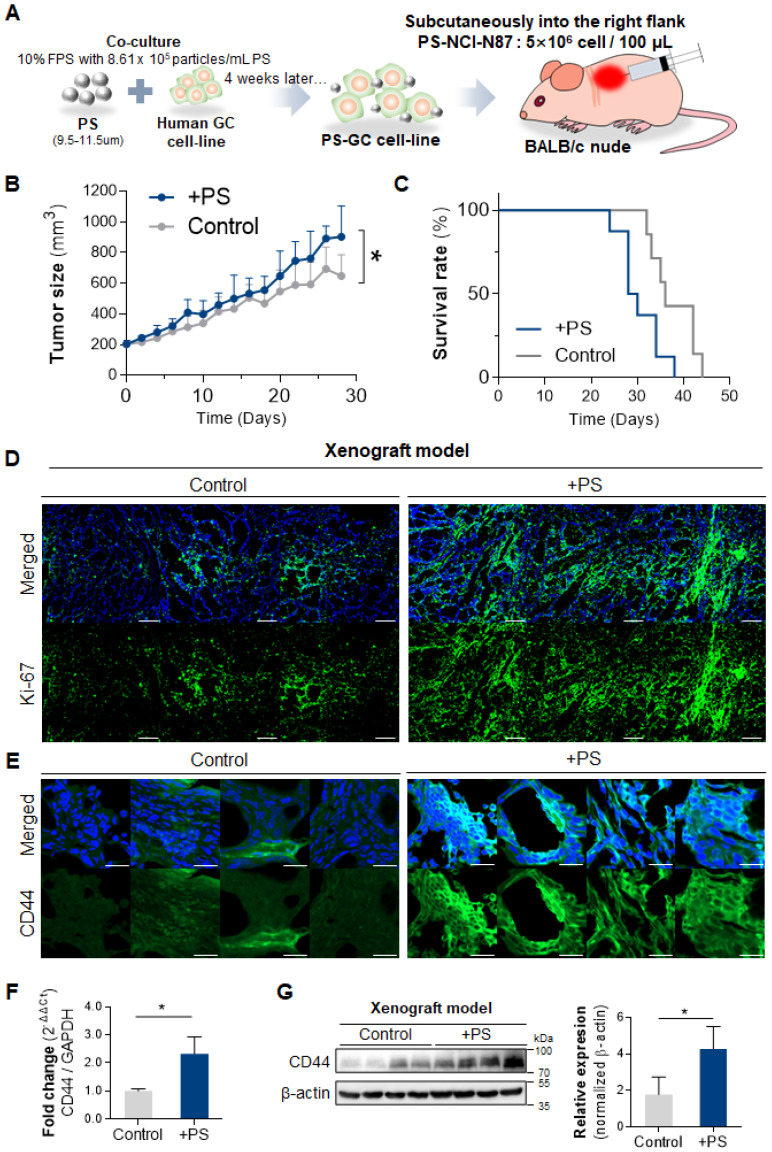
**Polystyrene accelerated tumor growth, decreased survival rate, and increased CD44+ cancer stem cell. (A)** Schematics of polystyrene (PS) exposure in a gastric cancer cell line and an *in vivo* model. The cells were exposed to 8.61 × 10^5^ particles/mL of PS during the subculture for 4 weeks. After 4 weeks, washed PS-exposed cells were used to generate a xenograft mouse model. **(B)** Tumor growth data for the PS-exposed NCI-N87 and NCI-N87 xenograft mouse models (*P* value: 0.0146, n = 7-8 mice in each group). **(C)** The survival rate data for the PS-exposed NCI-N87 and NCI-N87 xenograft mouse models (median survival: control, 36 days; PS-exposed NCI-N87, 29 days; log rank *P* value: 0.0210, n = 7-8 mice in each group). **(D)** Immunofluorescence of Ki-67 in NCI-N87 and PS-exposed NCI-N87 xenograft tissue. **(E)** Immunofluorescence images showing PS-exposed NCI-N87 tissue stained for CD44 (magnification, 40×; scale bar, 20 µm). **(F)** qPCR analysis of *CD44* expression. mRNA expression in PS-exposed NCI-N87 tissue (mean ± SD, * P < 0.05, Student's t-test). **(G)** Western blot analysis of CD44 expression in PS-exposed NCI-N87 tissue. PS exposure increased CD44 expression levels (mean ± SD; * P < 0.05, Student's t-test).

To investigate whether PS exposure affected tumor development, tumor growth, and survival rate *in vivo*, we subcutaneously injected 4-week PS-exposed NCI-N87 gastric cancer cells into mouse (**Fig. 3A**). An acceleration in tumor growth (relative to control) was observed in the PS-exposed NCI-N87 model (* P < 0.05; n = 7-8, **Fig. 3B)**. Individual tumor growth pattern was shown in **Fig. S7.** Median survival decreased from 36 to 29 days in the PS-exposed NCI-N87 model (* P < 0.05, n = 7-8, **Fig. 3C**). Immunofluorescence (IF) analysis reveals that Ki-67 was highly expressed in PS-exposed NCI-N87 tissue more than control tissue (**Fig. 3D**).

The tissue IF data reveal that CD44 expression was increased (compared with control) in PS-exposed mouse (**Fig. 3E**). The qPCR results demonstrate a 2.30-fold increase in *CD44* expression in the PS-exposed group (**Fig. 3F**). The western blot confirmed that CD44 protein expression increased 2.40-fold (compared to control) in PS-exposed mouse (**Fig. 3G**).

**Figure 4:**
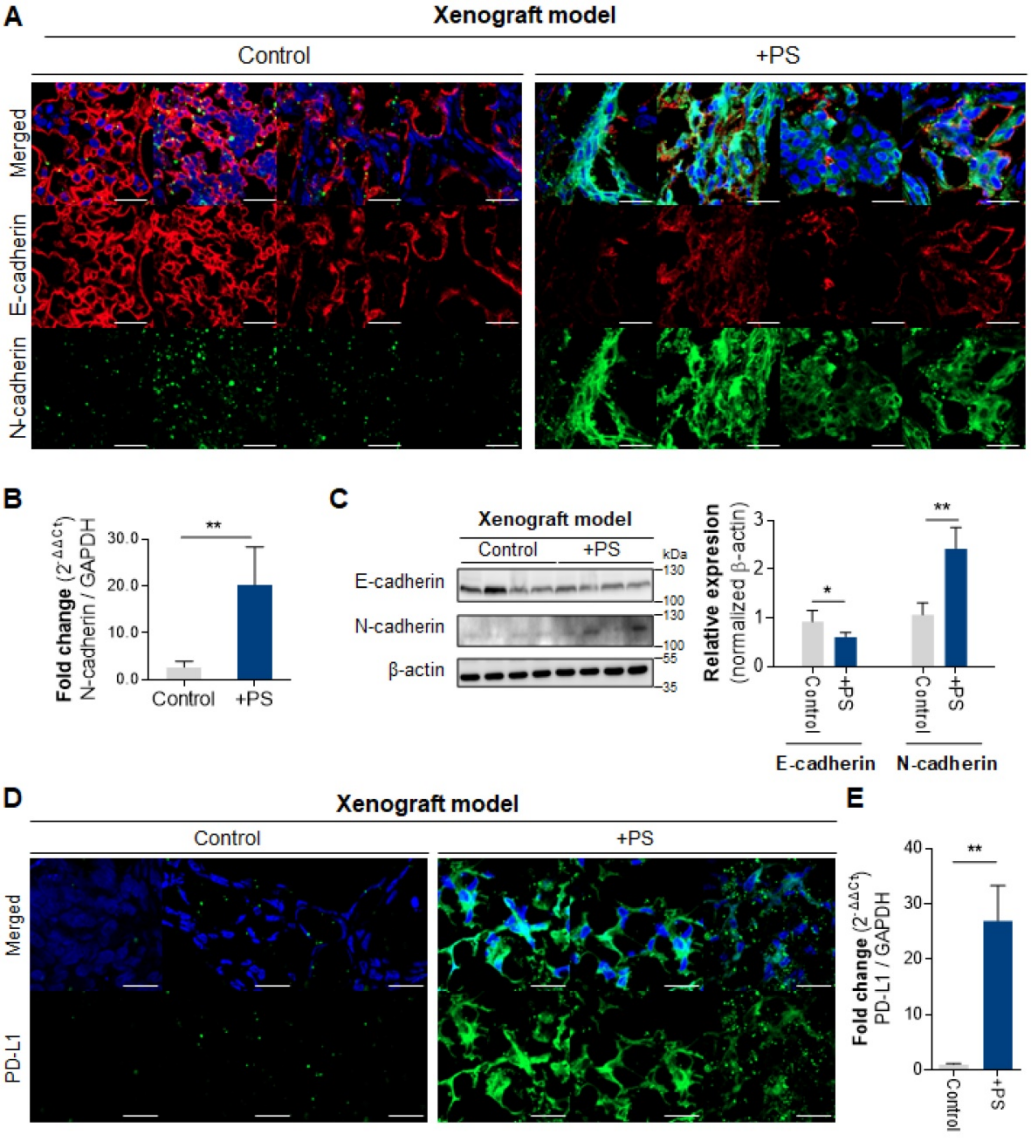
**Polystyrene exposed cell xenografted mouse model. (A)** Immunofluorescence images showing PS-exposed NCI-N87 tissue stained for E-cadherin and N-cadherin (magnification, 40×; Scale bar, 20 µm). **(B)** qPCR analysis of N-cadherin mRNA expression (mean ± SD, **P < 0.005, Student's t-test; n.s., not significant). **(C)** Western blot analysis of N-cadherin and E-cadherin expressions in PS-exposed NCI-N87 tissue. PS exposure decreased E-cadherin expression and increased N-cadherin expression (mean ± SD, * P < 0.05, ** P < 0.005, Student's t-test). **(D)** Immunofluorescence staining of PD-L1 in PS-exposed NCI-N87 tissue (magnification, 40×; scale bar, 20 µm). **(E)** qPCR analysis of PD-L1 mRNA expression in PS-exposed NCI-N87 tissue. (mean ± SD, **P < 0.005, Student's t-test).

The IF data demonstrated increased N-cadherin and decreased E-cadherin in the PS-exposed NCI-N87 group compared with control (**Fig. 4A**). The qPCR results demonstrate a 7.35-fold increase in N-cadherin expression in the PS-exposed group (**Fig. 4B**). Consistently, the western blot showed a 2.42-fold increase of N-cadherin (* P < 0.05), and a 0.61-fold decrease of E-cadherin (** *P* < 0.005, **Fig. 4C**).

The IF data reveals that PD-L1 expression was increased (compared with control) in PS-exposed NCI-N87 mouse model tissue (**Fig. 4D**). The qPCR results also demonstrate an increase in PD-L1 gene expression in PS-exposed NCI-N87 mouse (**Fig. 4E**).

### Differentially expressed genes after PS exposure

**Figure 5:**
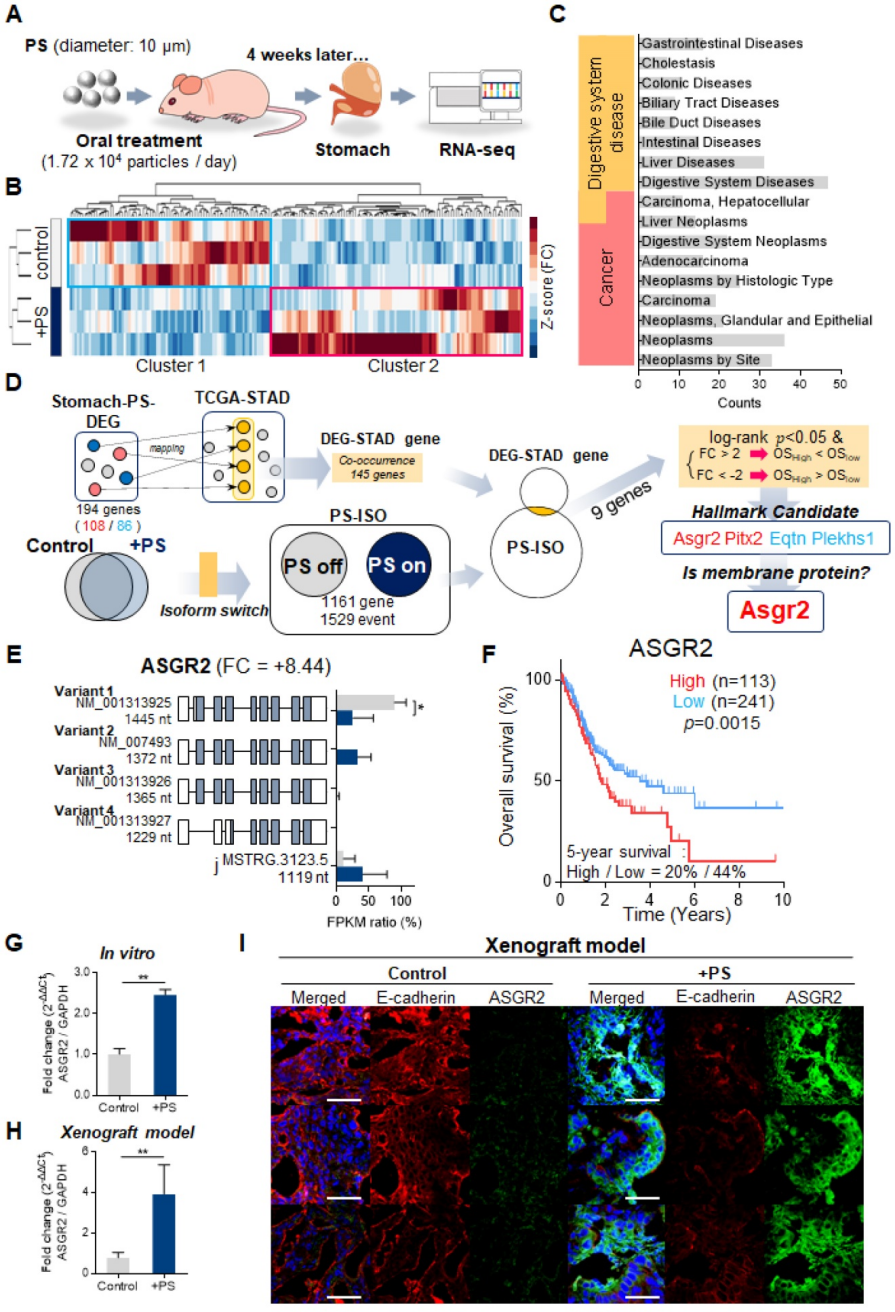
**Identification of ASGR2 using RNA-seq. (A)** Schematic of PS exposure in the *in vivo* model. BALB/c nude mice were exposed to PS (1.72 × 10^4^ particles/mL); 10 µm diameter) for 4 weeks. The stomachs were harvested from the PS-exposed mice, and total RNA was isolated. RNA-sequencing was then performed. **(B)** The result of hierarchical clustering analysis in differentially expressed genes (DEGs) (cut-off: 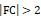 and * P < 0.05). The clustergram demonstrates the genes that were changed by PS exposure (blue, Cluster 1: gene decreased by PS; red, Cluster 2: gene increased by PS). DEGs were Z score corrected for display (relatively low expression is shown in blue and high expression in red). See also **Table S1**. **(C)** Analysis of the CTD database (https://ctdbase.org/) enrichment for collection of diseases in the DEG list. (The data cut-off p<0.01) **(D)** Schematic of the isoform switching method with differentially expressed genes (DEGs) and survival analysis. “PS-on” is defined as an increase in gene isoform ratio in the control following PS exposure. Conversely, “PS-off” is defined as a decrease in gene isoform ratio in the control following PS exposure (See also **Table S3**)**.** Nine genes were identified from the switch genes and DEGs. Next, 9 genes were investigated for their progressive effect (in upregulated genes) or suppressive effect (in downregulated genes) on the overall survival of genes in TCGA-STAD (TCGA-STAD-OS). Four genes were identified. Finally, we selected the membrane protein gene, *ASGR2,* based on our experiment data. **(E)** Five analyzed isoforms of the *ASGR2* gene after PS exposure. Variant 1 isoform expression ratio decreased after PS exposure (* p<0.01, PFAM domains are indicated in color. UCSC gene transcript IDs are shown for each isoform). **(F)** Effects of ASGR2 on 5-year overall survival (OS) in The Cancer Genome Atlas Stomach Adenocarcinoma (TCGA-STAD) datasets. TCGA-STAD datasets with high ASGR2 expression showed a tendency to be associated with poor 5-year OS (P = 0.0015, P-value: log-rank t-test). **(G-H)** qPCR analysis of ASGR2 mRNA expression in PS-exposed cell-line and NCI-N87 tissue. (mean ± SD, *P < 0.05, **P < 0.005, Student's t-test). **(I)** Immunofluorescence staining of ASGR2 in PS-exposed NCI-N87 tissue (magnification, 40×; scale bar, 20 µm).

Next, we investigated whether PS exposure induced gene expression changes in mice gastric tissue. To evaluate the changes in RNA expression following PS exposure, 10 µm PS (1.72 × 10^4^ particles/mL) was administered to BALB/nude mice (5-week-old, male; n = 3) daily for 4 weeks. After 4 weeks, we isolated the stomach and performed RNA-seq (**Fig. 5A**).

The Differentially expressed gene (DEG) analysis of mouse gastric tissue after PS exposure for 4 weeks was shown in **Fig. 5B**. In total, 194 genes showing significantly altered expression (cut-off fold change > 2; * P < 0.05) were identified in the PS exposure group when compared with the control group (**Fig. 5B** and **Table S1**). The prolonged administration of PS interacted with gastric tissue and altered the RNA expression. Enrichment analysis using CTD (comparative toxicogenomics database, cut-off *P < 0.05) was then used to identify genes in disease sets (**Fig. 5C**). Among the DEG gene sets, the strongest associations were seen in digestive system diseases (such as gastrointestinal disease, colonic diseases, and intestinal disease) and cancer (such as digestive system neoplasms and adenocarcinoma). This result can be presumed to be related to cancer. Further, gene expression change is presumed to be related to gastric cancer or disease after PS exposure.

### PS exposure disturbed RNA isoform distribution

A change in the RNA isoform distribution was identified in all samples. The event counts for isoform changes were listed in **Table S2**. The ratio of the sum of Fragments Per Kilobase of transcript per Million mapped reads (FPKMs) of gene isoforms (for genes with isoforms) was calculated using the switch method (**Fig. S8A**, cut-off * *P* < 0.05) 22. Finally, 1162 genes with 1529 events were identified (**Fig. S8A, Table S3**). Here, “PS-on” was defined as an increase in gene isoform ratio in the control following PS exposure. Conversely, “PS-off” was defined as a decrease in gene isoform ratio in the control following PS exposure. The ΔFPKM ratio (ΔFR) was analyzed (ΔFR = [FPKM_PS_]_avg_ - [FPKM_control_]_avg_). Using ΔFR > 0, 495 multi-exons with at least one junction match (j), six containment of reference (reverse containment) events (k), zero single exon trasfrags partially covering an intron, and possible pre-mRNA fragment events (e) were observed (**Table S4**). The number of complete events that appeared was 377 (**Table S4**). Using ΔFR < 0, 357 state j, four state k, and one state e were observed (**Table S4**). The number of complete events that appeared was 301 (**Table S4**). The distribution of ΔFR change for each gene is displayed using a volcano plot (**Fig. S8B**). Representative genes showing changes in ΔFR > 0.8 and ΔFR < -0.8 are shown in **Fig. S8C**. These results show that PS exposure disturbed the RNA isoform change in gastric tissue.

### Identification of ASGR2

Co-occurrence genes (named DEG-STAD gens) between TCGA-STAD and DEG was identified. The genes with isoform switch were identified (named as PS-ISO including PS on, PS off). Then co-occurrence genes between PS-ISO and DEG-STAD were identified (**Fig. 5D,** |FC|>2& p<0.05). Then 9 genes (6 upregulated and 3 downregulated) were identified. Among these genes, *ASGR2*, pituitary homeobox 2 (*PITX2*), equatorin (*EQTN*), and pleckstrin homology domain-containing family S member 1 (*PLEKHS1*) genes were related with malignancy in TCGA-STAD patients. Although nano-sized particles 23 or < 3 μm sized MP 24 were internalized by cells, there was no evidence of cell penetration for 10 μm sized PS, which was used in our study (**Fig. 1D**). Therefore, among 4 genes, we finally selected membrane protein ASGR2 as a gene of interest.

### PS exposure increased ASGR2

For *ASGR2*, the mean expression ratio of variant 1 (NM_001313925, Product size: 1445 nt) was 89.53% in the PS-off state (*P < 0.05, **Fig. 5E**). In the PS-on state, the mean expression ratio of variant 1 decreased to 25.64% (ΔFR = -63.89, * P < 0.05, **Fig. 5E**). Interestingly, the FPKM ratio of variant 1 decreased; however, gene expression increased by 8.44-fold. According to the TCGA-STAD datasets and 5-year OS associated with *ASGR2*, high *ASGR2* expression was associated with poor 5-year OS (log-rank *P-*value = 0.0015, **Fig. 5F**).

Next, we investigated whether PS exposure induced ASGR2 expression changes *in vitro* and *in vivo*. The qPCR results demonstrated a 2.45-fold increase in *ASGR2* expression in PS-exposed NCI-N87 cells (**Fig. 5G**). Increased *ASGR2* expression after PS exposure was also observed in every cell line (**Fig. S8D**). In addition, the qPCR results demonstrated a 4.60-fold increase in *ASGR2* expression in the PS-exposed xenografted group (**Fig. 5H**). The tissue IF data revealed that ASGR2 expression increased, and E-cadherin expression decreased (compared with that in control) in PS-exposed mice (**Fig. 5I**).

### Inhibition of ASGR2 using siASGR2

**Figure 6:**
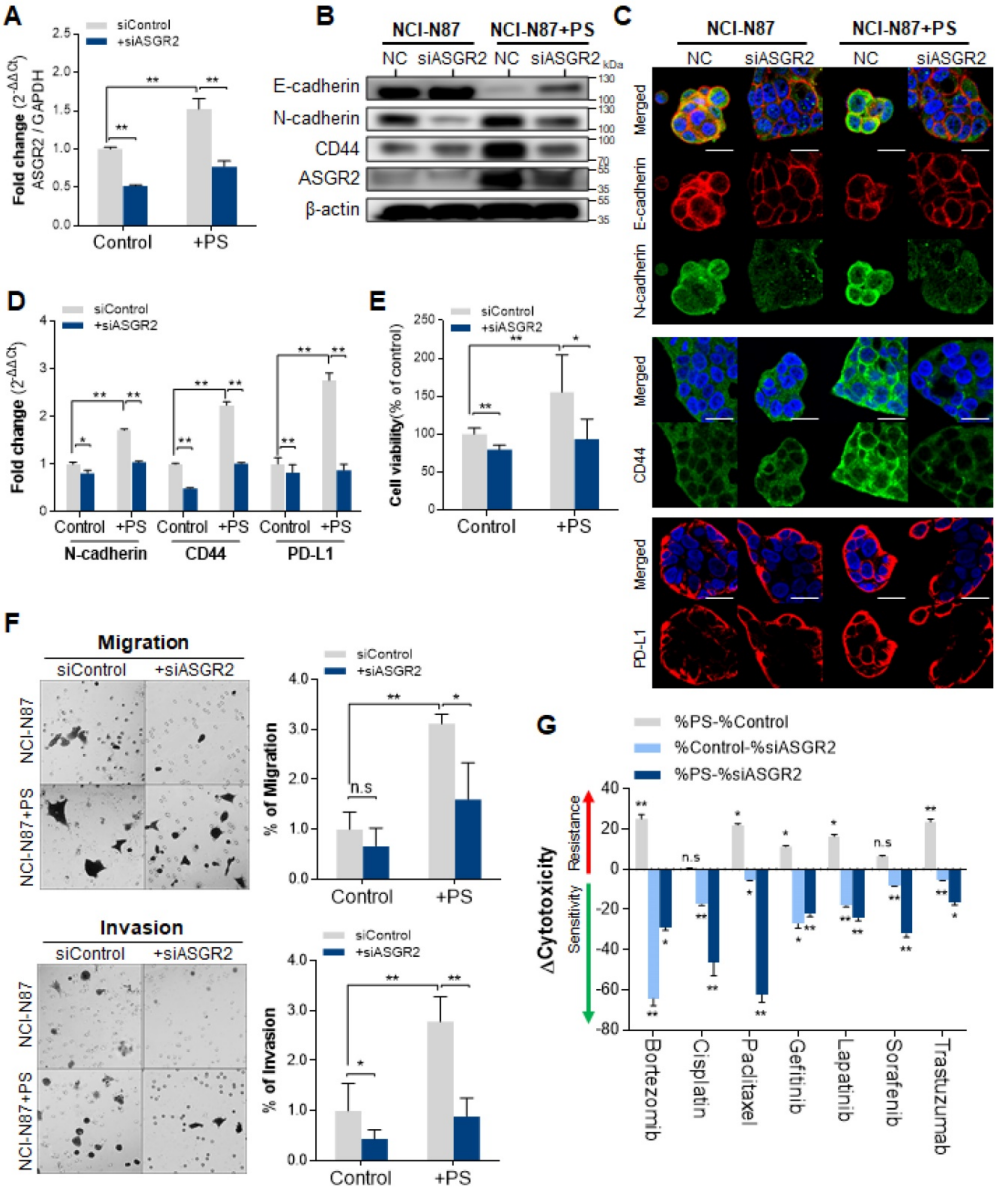
**Inhibited effect of ASGR2. (A)** Validation of ASGR2 knockdown via siRNA in NCI-N87 cells (mean ± SD, * P < 0.05, ** P < 0.005, Student's t-test). **(B)** Western blot analysis of the expression of cancer hallmarks in PS-exposed NCI-N87 cells with ASGR2 knockdown. **(C)** Immunocytochemistry staining showing N-cadherin, E-cadherin, CD44, and PD-L1 expression in NCI-N87 cells with ASGR2 knockdown (magnification, 40×; scale bar, 20 µm). **(D)** qPCR analysis of N-cadherin, CD44, and PD-L1 mRNA expression in PS-exposed cell lines with ASGR2 knockdown (mean ± SD, *P < 0.05, **P < 0.005, Student's t-test). **(E)** Validation of ASGR2 knockdown for proliferation. **(F)** *In vitro* migration and invasion assays. Bar graphs represent the average number of cells on the underside of the membrane normalized to the control condition. siASGR2 suppressed the migration and invasion of NCI-N87 cells (magnification, 20×; mean ± SD; * P < 0.05, ** P < 0.005, n.s., not significant, Student's t-test). **(G)** ASGR2 mediated drug resistance after PS exposure (10 µm diameter; 8.61 × 10^5^ particles/mL; 4 weeks). The cytotoxicity of bortezomib, cisplatin, paclitaxel, gefitinib, lapatinib, sorafenib, or trastuzumab were evaluated. “Δcytotoxicity = %cytotoxicity w/ PS - % cytotoxicity w/o PS”, “%cytotoxicity w/o PS - % cytotoxicity w/ siASGR2”, or, “%cytotoxicity w/ PS - % cytotoxicity w/ siASGR2” were measured based on quantitative analysis. If Δcytotoxicity > 0, then “drug resistance”, and If Δcytotoxicity < 0, then “drug sensitivity” (* P < 0.05, ** P < 0.005, n.s., not significant. Student's t-test).

The inhibition effect of ASGR2 was investigated. The qPCR results confirmed that siASGR2 suppressed *ASGR2* expression by 0.52-fold compared with that in negative siRNA control (**Fig. 6A**). After confirming ASGR2 expression was increased by PS, then we confirmed that siASGR2 significantly suppressed ASGR2 expression by 0.77-fold (**Fig. 6A**). Next, we investigated whether the knockdown of ASGR2 affected N-cadherin, CD44, or PD-L1 expression. Western blotting confirmed that ASGR2 protein expression increased (compared with that in control) in PS-exposed NCI-N87 cells. Simultaneously, CD44 and N-cadherin expression increased, and E-cadherin expression decreased (compared with that in control) in PS-exposed NCI-N87 cells (**Fig. 6B**). Next, western blotting confirmed that ASGR2 was reduced (compared with that in control) in siASGR2-treated NCI-N87 cells or siASGR2 plus PS-treated NCI-N87 cells (**Fig. 6B**). Decreased CD44, PD-L1 and N-cadherin expression, and increased E-cadherin expression was observed (compared with that in control) in PS-exposed NCI-N87 cells treated with siASGR2 in western blot (**Fig. 6B**) and ICC (**Fig. 6C**).

In addition, the qPCR results show that a 1.71-fold increase in N-cadherin gene expression after PS exposure reduced to 1.04-fold after siASGR2 treatment. In addition, a 2.24-fold increase in CD44 gene expression after PS exposure reduced to 1.01-fold after siASGR2 treatment (**Fig. 6D**). Further, a 2.76-fold increase in PD-L1 gene expression after PS exposure reduced to 0.87-fold after siASGR2 treatment (**Fig. 6D**). These patterns were also similarly observed in other cell lines (**Fig. S9A**-**B**)**.** These results show that ASGR2 can control N-cadherin, CD44, and PD-L1 gene and protein expression.

### Knockdown of ASGR2 inhibited proliferation, migration and invasion, and multidrug resistance

We investigated whether PS induced cell proliferation through ASGR2. After siASGR2 treatment, cell proliferation was significantly suppressed from baseline to 79.56% in control NCI-N87 cells (**P<0.005, **Fig. 6E**) and from 155.6% to 96.68% in PS-exposure NCI-N87 cells (*P<0.05, **Fig. 6E**). Moreover, these patterns were also observed in other cell lines (**Fig. S9C**)**.** These results show that PS exposure induces cell proliferation through ASGR2.

Next, we investigated whether PS induced migration and invasion through ASGR2. The cell migration assay results showed that siASGR2 significantly suppressed migration rate from 3.12-fold to 1.6-fold in the PS-exposure group (*P<0.05, **Fig. 6F**). The cell Invasion assay results showed that invasion rate was significantly suppressed by 0.44-fold after siASGR2 treatment in NCI-N87 cells (*P<0.05, **Fig. 6F**). siASGR2 significantly suppressed invasion rate from 2.79-fold to 0.89-fold in the PS-exposure group (*P<0.05, **Fig. 6F**). These patterns were observed in other cell lines (**Fig. S9D**)**.** These results confirmed that ASGR2 increased migration and invasion rate.

Next, we investigated whether PS reduced drug resistance through ASGR2. siASGR2 increased drug sensitivity in normal NCI-N87 cells. Specifically, proliferation was strongly suppressed to -64.45% by bortezomib treatment compared with that of control (**Fig. 6G**). siASGR2 strongly suppressed proliferation together with other drugs.

In addition, siASGR2 decreased PS-induced drug resistance and increased drug sensitivity. Proliferation was strongly suppressed from 25.15% to -29.03% by bortezomib treatment, from 22.03% to -62.40% by paclitaxel treatment, and from 23.56% to -16.49 by trastuzumab treatment. siASGR2 also similarly increased to other drugs after PS exposure (**Fig. 6G**). These results show that ASGR2 mediated the enhancement of proliferation, migration and invasion, and multidrug resistance after PS exposure.

## Discussion

**Figure 7:**
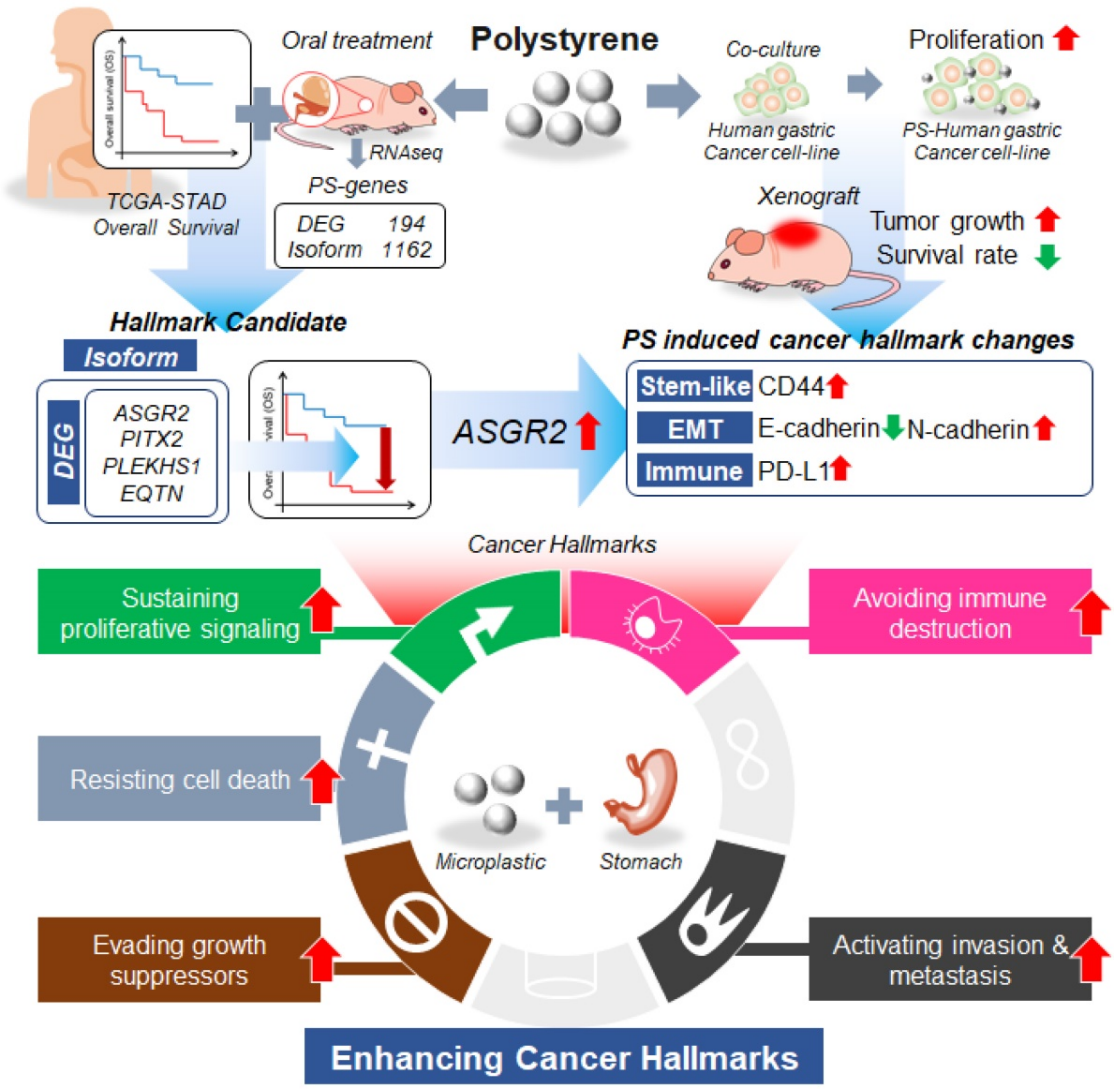
**Summarized results of the study.** We showed that microplastic polystyrene exposure enhanced invasion and migration, drug resistance, and stem-like properties after PS exposure. We examined using RNA sequencing whether polystyrene exposure perturbed cancer-associated gene expression. We identified that the expression level of asialoglycoprotein receptor 2 (ASGR2) was increased after prolonged exposure of polystyrene. We demonstrated ASGR2 as a possible oncogene in gastric cancer.

We showed that PS exposure causes ASGR2 gene alterations associated with gastric cancer. To our best knowledge, it is the first study on the effect of MP to cancer risk at *in vivo* level. In this study, we showed increased tumor cell proliferation and increased tumor volume after 4 weeks exposure of 10 μm sized PS. Increased cell proliferation was shown using 5 different gastric cancer cells including NCI-N87, AGS, MKN1, MKN45, and KATOII. We examined the correlation of changes in gene expression observed in patients with those in published datasets such as TCGA-STAD and demonstrated that PS exposure leads to a change in the pattern of RNA isoform expression (**Fig. 7**).

Until now, the risk of MP to gastric cancer was unclear and unknown. MP as a potential immune stimulants that induced cytokine and chemokine production were reported at the cellular level 25, which was not *in vivo* level. MP can carry a range of contaminants such as trace metals and some potentially harmful organic chemicals 26. Capture of carcinogenic polycyclic aromatic hydrocarbons onto MP by adsorption phenomena and an assessment of probable cancer risk of ingested polycyclic aromatic hydrocarbons enriched MP by human beings was reported 27. However, the study 27 evaluated the effects of plastic adsorbents, not the plastic itself revealed a link to cancer. Here, we demonstrated that MP exposure is a novel risk factor for gastric cancer.

First, we identified if the PS remained in the stomach 24 hours after feeding on a single dose of [^64^Cu]Cu-DOTA-polystyrene **(Fig. 1B)** and 1.72 × 10^4^ PS particles /mL of PS exposure for 4 weeks provided evidence of its accumulation in the gastric tissue **(Fig. S1).** High MP concentrations were detected in the stools of inflammatory bowel disease patients when compared to those of a healthy person 28. Interaction between MP and *Helicobacter pylori* contributed to the rapid bacterial colonization of gastric mucosal epithelial cells, improved the efficiency of MP entry into tissues, and promoted gastric injury and inflammation in mice 29. Recently cytotoxicity of PS was reported for human dermal fibroblasts, human mast cell line, peripheral blood mononuclear cell, which was focused on immune system through skin exposure 25. Our result and ref 25, 28 demonstrated that exposure of MP could affect not only exacerbation of cancer but also immune response 25.

In the real world, MP was found to be ubiquitous across ecosystems, and its annual consumption was found to range from 74,000-121,000 particles 30. In previous studies, on the mechanisms of toxicity, the MP concentrations ranged from 2.27 × 10^4^ particles/mL to 1.46 × 10^6^ particles/mL 31, 32. Considering this, the exposure level used in this study was determined to be 1.72 × 10^5^ PS particles/mL to 8.61 × 10^5^ PS particles/mL. The PS concentrations in this study were relatively high when compared to real world MP exposure, since, we aimed to reveal the possible toxicity of MP to organs and longitudinal exposure of MP to gastric tissues. Furthermore, environmental pollution is accelerating, and plastic use increasing. Thus, while the amount of MP exposure used in this study is greater than current real world exposure levels, it is suggested that continuous and repetitive MP exposure is likely to cause ASGR2 gene alterations related to gastric cancer, as presented in this study.

Next, we consider the problem of MP transition. MP was potentially digested to small fragments by high acidic gastric conditions (pH 1-3) 29, 30. Larger MP particles can be digested to fragments in the digestive track after intake 29, 33. According to our previous results, PS began its transit to the intestine within 1 hour of consumption and was then transited to organs such as the spleen, liver, heart, lung, kidney, and bladder 16. Recently, we revealed that fragmented MP could be transited to the brain, and induce an autism spectrum disorder 8. We may predict similar result for MP exposed to the stomach due to the MP environment when MP is continuously exposed to the stomach, fragmented by gastric acid, suggesting that the finely fragmented MP could be transited to human organs including the brain.

RNA-seq has become an indispensable tool for transcriptome-wide analysis of differential gene expression and differential splicing of mRNAs 34. Using RNA-seq, we revealed that PS exposure disturbed gene expression, and isoform distribution, within gastric tissue. A recent RNA-seq analysis assessed the global change in gene expression caused by PS in *Daphnia pulex*, a model organism for ecotoxicity 35. However, there was no result to reveal the potential risk associated with stomach exposure to MPs from ref 35. Using RNA-seq, we identified enhanced *ASGR2* oncogene on stomach after prolonged PS exposure. Therefore, we selected ASGR2 as a gene of interest. We showed ASGR2 enhanced typical cancer hallmark. In addition, knockdown of ASGR2 reduced PS-induced enhanced migration and invasion rates. Enhanced in ASGR2 were maintained at *in vivo* level. ASGR2 knockdown resulted in the upregulation of CD44 and drug resistance recovery. ASGR2 accelerated the tumor growth. Based on the result, PS exposure in patients with gastric cancer possibly causes resistance to chemotherapy and drug therapy. The roles of ASGR2 were confirmed from other studies. Increased ASGR2 was closely associated with poorer prognoses, EMT, and proliferation in gastric cancer 36. Transcriptomics Identified ASGR2 as a predictor of hematogenous recurrence of gastric cancer 36. Using the mouse hepatic metastasis model, ASGR2 knockdown was shown to suppress metastasis strongly 36. When comparing the metastases to intact liver, up-regulated ASGR2 gene was contributed by the host tissue 37. Hazardous substances, such as endocrine-disrupting chemicals, benzo(a)pyrene, aflatoxin B1, methapyrilene 38, and carbon black nanoparticles 39, cause increased ASGR2 expression.

In this study, we found 1161 genes were changed in the pattern of isoform after PS exposure. Generally, in cancer, isoform change was correlated with EMT, drug resistance, and stem cell differentiation 22, 40, 41. CD44 expression was enhanced, N-cadherin expression, invasion, and migration were increased. Long-term exposure to PS induced an immunosuppressive environment in gastric cancer tissues. PD-L1 expression was also increased after PS exposure.

In our study, although we tested five gastric cancer cell lines including NCI-N87, AGS, MKN1, NKN45, and KATOIII, we focused on NCI-N87 as a cell of interest. This was because we have future plans to investigate PS induced drug resistance for radioimmunotherapy (RIT) 42. Recently, targeted therapies using therapeutic technologies, have been widely used in clinics. Especially as RIT was introduced as an emerging technology. RIT uses radioisotope conjugated with monoclonal antibody (mAb) in the treatment of cancer. RIT, mAb labeled with radionuclides, is an appealing procedure for cancer treatment because tumor linked-mAb with cytotoxic radionuclides can selectively bind to tumor antigens 42. Human epidermal growth factor receptor 2 (HER2) is a tyrosine kinase receptor involved in the pathogenesis of several cancers, including advanced gastric and gastroesophageal junction cancer. HER2 protein binds to an extracellular ligand binding domain that initiates a signal transduction cascade that affects tumor cell biology through several mechanisms, including cell proliferation, apoptosis, adhesion, migration, and differentiation. Trastuzumab (TZB) (Roche, USA) is the first humanized mAb approved for immunotherapy and the first oncogene-targeted treatment with proven survival benefit in HER2-positive cancer 43. NCI-N87 cell is a HER2-positive gastric cancer cell line. However, according to our preliminary western blotting data, Her2 expression was null or very low in the AGS, MKN1, NKN45, and KATOIII cell lines (**Fig. S10).** Western blotting confirmed that Her2 expression did not change after PS exposure **(Fig. S10).**

For further study, the reason of increased ASGR2 after PS exposure need to be revealed. One of possible reason was the mechanical force. The Nestor-Bergmann et al. study showed that mechanical forces, such as stretching on tissues, tension, and compression, were associated with division rate 44. The proliferation rate was regulated by mechanical stress, correlated with relative isotropic stress in the *Xenopus* tissue model 44. Extensive *in vitro* studies on mechanical stress showed that malignant phenotypes in non-malignant cells were promoted 45. There are many more examples of processes (not only during development) where the presence of mechanical stress has been inferred from experimental approaches, such as birefringence measurements 46 or by observation of the geometry of the cell shapes in the tissue 47. However, in our experimental condition, we just mixed PS and culture media. Therefore, it was unclear how much of “mechanical force” due to PS could be generated by simple *in vitro* mixing in the media. Another possible reason is PS penetration into cell. Plain PS is a hydrophobic particle with no hydrophilic functional group such as -OH, -NH_2_, or -COOH. Generally, the cell membrane and membrane proteins are hydrophilic. Although there was a report on cell penetration of 20 nm sized PS nanoparticles (carboxylate, sulfate, or aldehyde-sulfate modified [negatively charged] and amidine-modified [positively charged]) across rat alveolar epithelial cell monolayers 23, there was no evidence of cell penetration for 10 μm sized plain nonfluorescent PS with no hydrophilic functional group, which was used in our study. Therefore, possibly there was no effect on cellular internalization of PS.

In this study, we have shown that PS induces drug resistance for multiple drugs including gefitinib, herceptin, sorafenib, cispatin, lapatinib, paclitaxel, and bortezomib. Briefly, gefitinib was used as an EGFR inhibitor 48, trastuzumab as a Her2 inhibitor 49, Lapatinib as an EGFR & Her2 inhibitor 50, sorafenib as a multikinase inhibitor 51, bortezomib as a protasesome inhibitor, and parclitaxel as a cytoskeletal inhibitor. We demonstrated that PS induced resistance to the therapeutic efficacy of multiple drugs in our study in gastric cancer cell lines (**Fig. 2J**). We showed that cancer stemmess (CD44+ in this study) 18, 19, 52, 53 increased by PS exposure through ASGR2. Increased CD44+ in gastric cancer induced cisplatin resistance due to the activation of the Hedgehog pathway 53. CD44 can be bound directly to EGFR or HER2 52. This interaction leads to the activation of MAPK signaling and could induce drug resistance in gastric cancer 52. Multidrug resistance (MDR) protein or ABC-type transporters cause bortezomib or parclitaxel drug resistance 20, 54. The increased level of CD44 expression caused an increase in MDR expression 55. Thus, drug resistance of parclitaxel may possibly be due to increases in MDR1 due to PS exposure through the CD44 pathway.

MP monitoring in the environment is crucial to determine the relevance and trends in MP contamination and plan environmental protection policies 56. Previously, researchers compared the characteristics of MPs using micro-ATR-FTIR spectra 56. In this study, we identified the possible toxicity in the gastric tissue using PS. In the preliminary study, we compared possible toxicity using polyethylene (PE) by measuring the expression of E-cadherin, N-cadherin, CD44, and PD-L1 via IF, qPCR, and western blotting (**Fig. S11).** We found that there were no significant differences between the PE-exposed group and the control group. Although we showed the preliminary results of how PE affected the cells, we could not conclude that there was no effect on the gastric cell lines due to density changes and cellular contact. As the density of PS was more than 1 g/cm3, it naturally covered the cell culture dish surface. PS easily interacted with the attached cells. However, in the case of PE, its interaction with the cells would be difficult because the PE particle density was less than 1 g/cm3. In addition, the probability of the contact between PE and cells was close to 1% even at high concentrations of PE (5000 ppm) 57. In a previous report, the PE-cell interaction challenge was identified 58, 59. They described that PE floats on the surface of the solution 58, 59, making cellular contact quite challenging. To resolve this, a surfactant buffer, such as Tween 20 or 80, was previously used with the PE, as it enforces an interaction between PE and cells 58, 60, 61. However, surfactants could induce cytotoxicity and increase the contact probability of PE with the cell 57, 58. Therefore, our study focused on using PS particles only. Exposure to PE would have confounding effects if a surfactant buffer was added.

In our study, to compare the effect of *in vitro* doses, three concentration groups, 0.86 × 105, 1.72 × 105, and 8.61 × 105 particles/mL, were compared. We determined how the expression of E-cadherin, N-cadherin, and CD44 changed according to the doses. We identified that there were no differences in the dose effect between 1.72 × 105 and 8.61 × 105 particles/mL (Fig. S4). The reason for using 8.61 × 105 particles/mL in the *in vitro* study was to increase the probability of contact between cells and PS, and to induce acute effects. As there was no dose effect between 1.72 × 105 and 8.61 × 105 particles/mL, we used 1.72 × 105 particles/mL for the *in vivo* study to minimize potential unexpected effects. One possible effect includes decreased bioavailability of lipid droplets by forming large lipid-MP heteroaggregates owing to the high MP hydrophobicity 62. The dose determination for *in vitro* and *in vivo* experiments should be considered in future studies. Mathematical modeling has been attempted to bridge the gap between *in vivo* and *in vitro* doses 63-65.

In summary, we showed MP exposure would be a new threat to gastric cancer. Especially, we identified ASGR2 as the messenger of PS-mediated changes in cancer hallmarks.

Our preclinical findings may provide an incentive for further epidemiological studies examining the role of MP exposure in the incidence of gastric cancer. If we can detect MPs from the stomach of patients and reveal the correlation between the presence of MP and the incidence of gastric cancer, it might be definite evidence for the risk associated with MPs. However, this would be limited because sampling of the normal gastrointestinal tissue in patients is currently an experiment that is not entirely ethical, and it is necessary to develop advanced technologies to detect MPs in gastric cancer tissues from patients.

## Material and methods

### Cell lines and PS exposure *in vitro* model

The human gastric cancer cell lines, AGS, MKN1, MKN45, and KATO III, were obtained from the Korea Cell line Bank (KCLB, Rep of Korea). NCI-N87 was obtained from the American Type Culture Collection (ATCC, USA). All cell lines were maintained in RPMI 1640 medium (Corning, USA) supplemented with 10% fetal bovine serum (FBS) and 1% penicillin/streptomycin at 37 °C in 5% CO_2_.

In previous studies, to reveal possible underlying mechanism during MP exposure, high doses of MP (2.27 × 10^4^ particles/mL to 1.46 × 10^6^ particles/mL) were exposed to the cells, tissues, and organs 25, 29, 66. The concentrations of polystyrene (PS) used to dose the cells were based on similar doses which were described in previously published articles 25, 29, 66. PS (500 mg, Diameter 9.5-11.5 µm, Cospheric, USA) were used to evaluate the effects of MPs on gastric cancer cells. To create a 5% PS suspension, ultrasonic grinding and vortexing were performed repeatedly. The 5% PS suspension was directly added to the cell culture media during subculture to reach the desired final concentration, 8.61 × 10^5^ PS particles/mL The PSs were removed by washing three times with PBS.

### Estimation of the number of PS particles

The number of PS particles was determined using Countess II^®^ (Invitrogen, Waltham., MA USA) according to the manufacturer's instructions and Kim, Jeong 31. Five hundred milligrams of PS was mixed with 10 mL of normal RPMI culture medium containing 10% FBS and 1% antibiotics. Thereafter, the samples were sonicated for 1 min and vortexed repeatedly. Subsequently, 0.1 mL of the mixture was stained with trypan blue solution (1:1 volume). The stained sample was loaded on Countess™ Cell Counting Chamber Slides (Invitrogen, USA) and read using Countess II^®^.

### Animal care

All animal experiments were performed under the institutional guidelines of the Korea Institute of Radiology and Medical Sciences (KIRAMS). All protocols were approved by the institutional Animal Care and Use Committee (IACUC number: KIRAMS 2020-0015). BALB/c nude mice (Shizuoka laboratory center, Japan) were 5 to 6 weeks old at the start of the experiments. The animals were weighed, caged, housed, and maintained for 24 h (under 12 h light/dark cycle).

### PET imaging of microplastic transition to stomach

We identified the absorption path and distribution of MPs using Positron emission tomography 16. Briefly, MP PS was labeled with Copper-64 ([^64^Cu], to yield [^64^Cu]Cu-DOTA-polystyrene), and then orally administered to mice. ^64^Cu-DOTA-PS (130 μCi/100 μL) was orally administered to 5-week-old BABL/c nude mice (male, n = 5).

### Animal model for microplastic accumulation in gastric tissue

Fluorescent green PS (diameter 8.0-8.9 µm; Spherotech, USA) were used to identify MP accumulation in gastric tissue. We orally administered 1.72 × 10^4^ particles/mL to 5-week-old BALB/c nude mice (Shizuoka laboratory center, Japan) daily for 4 weeks. After 4 weeks, the mice were euthanized using CO_2_. The stomach was isolated and fixed overnight in 4% PFA at 4 °C. Frozen sections (8 µm thick) were generated using optimal cutting temperature (OCT) compound, and the sections were transferred to glass slides. Cell nuclei were stained with DAPI (ThermoFisher Scientific, USA), and images were obtained using confocal microscopy (LSM-880, Zeiss, Germany).

### Animal model for PS-exposed cell xenografted mouse model

Five-week-old male BALB/c nude mice (Shizuoka laboratory center, Japan) were maintained in temperature-controlled clean racks under a 12-h light/dark cycle. Mice were allowed to acclimatize for one week before the start of experiments. We injected 5 × 10^6^ NCI-N87 cells or 5 × 10^6^ PS (8.61 × 10^5^ PS particles/mL for 4 weeks)-exposed NCI-N87 cells subcutaneously into the right flank of these mice. The health status of the mice was then checked daily. When the tumor size reached approximately 200 mm^3^, the mice were assigned to a group (n = 7-8/group). The tumor size was measured at the indicated times using a caliper, and the tumor volume was calculated using the formula width^2^ × length × 0.5. All mice were euthanized when the tumor size exceeded a volume of 1,000 mm^3^.

### Measurement of proliferation

To investigate PS cytotoxicity, cell proliferation was measured using the alamarBlue® assay (ThermoFisher Scientific, USA) following the manufacturer's instructions. Briefly, cells were seeded onto 96-well culture plates at a density of 5,000 cells/well (using RPMI-1640 medium supplemented with 10% FBS), and subsequently treated with PS microspheres. Cell proliferation was measured at λ_em_ = 590 nm (λ_ex_ = 560 nm) using a microplate reader (i3X, Molecular Devices, USA). Background emission fluorescence Intensity (FI) was subtracted for each medium. Emission values were calculated as the percentage of control, yielding percentage cell proliferation 72 h after seeding as follows:



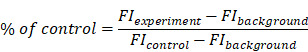



### Assessment of drug resistance

To assess drug resistance due to CD44, cytotoxicity was measured using bortezomib or cisplatin. The cytotoxicity was determined using the alamarBlue® assay (Thermo Fisher Scientific, Waltham, MA) following the manufacturer's instructions. Briefly, cells were exposed to PS for 4 weeks. The cells were then seeded in 96-well culture plates at a density of 1 × 10^5^ cells/mL in medium (supplemented with 10% FBS) containing 8.61 × 10^5^ particles/mL of PS. The cells were incubated overnight and then treated with: bortezomib (Takeda Oncology, USA, 10 nM in PBS); cisplatin (United states Pharmacopeia, USA, 1.5 μg/mL in dimethyl sulfoxide [DMSO]); paclitaxel (Sigma-Aldrich, USA, 300 nM in DMSO); gefitinib (Sigma-Aldrich, USA, 4 mM in DMSO); lapatinib (Sigma-Aldrich, USA, 1 μM in DMSO); sorafenib (Sigma-Aldrich, USA, 10 μM in DMSO); or trastuzumab (Roche, Switzerland. 10 μg/mL in PBS). All treatment incubations were performed in RPMI-1640 medium supplemented with 5% FBS and/or 8.61 × 10^5^ particles/mL of PS. Cytotoxicity was measured at an emission of 590 nm using a microplate reader. The emission values are reported as a percentage of vehicle control, yielding a percentage cell cytotoxicity after 72 h of treatment. For the calculation of drug resistance, Δcytotoxicity was calculated by followings; Δcytotoxicity = %cytotoxicity w/ PS - % cytotoxicity w/o PS.

### Flow cytometry

To detect N-cadherin, CD44, and PD-L1 expression, cells were exposed to PS for 4 weeks, dislodged with EDTA-trypsin, and then collected in 5 mL FACS tubes (1 × 10^6^ cells/tube). The cells were then centrifuged and the supernatant was aspirated. The cells were then incubated for 30 min in 4% paraformaldehyde at room temperature. After removing the fixation buffer, permeabilization buffer (0.2% Triton-X in distilled water) was added to the cells. After permeabilization, the buffer was aspirated and the cells were incubated with blocking buffer (1% BSA, 10% normal goat serum) for 1 h at room temperature. The cells were then incubated with primary antibody (anti-N-cadherin, anti-CD44, or anti-PD-L1 antibody, see also **Table S6**) overnight at 4 °C. After washing three times with PBS, the cells were incubated with Alexa Fluor-647 goat anti-rabbit IgG (H + L) at room temperature in the dark for 60 min. After, washing three times with PBS, antibody signals were detected using flow cytometry (BD Accuri™ C6 Plus, Becton Dickinson Biosciences, USA).

### Immunocytochemistry (ICC)

Briefly, the cells were seeded in a two-well chamber slide (Falcon, USA) in RPMI 1640 medium (Corning, USA) supplemented with 10% FBS at a density of 1 × 10^5^ cells/mL and then treated with 8.61 × 10^5^ particles/mL of PS. The cells were then incubated overnight at 37 °C in 5% CO_2_. After aspiration of the culture medium, the cells were washed three times with PBS and fixed with 4% paraformaldehyde for 20 min at room temperature. After fixation, the cells were permeabilized by incubation with 0.2% Triton-X in distilled water for 20 min at room temperature. After buffer aspiration, the cells were incubated with blocking buffer (1% BSA and 10% normal goat serum) for 1 h at room temperature. The cells were then incubated with primary antibody (anti-N-cadherin, anti-E-cadherin, anti-CD44, or anti-PD-L1, see also **Table S6**) overnight at 4 °C. N-cadherin and E-cadherin were detected with Alexa Fluor-488 goat anti-rabbit IgG (H + L) and Alexa Fluor-647 goat anti-mouse IgG (H + L), respectively. PD-L1 was detected with Alexa Fluor-647 goat anti-rabbit IgG (H + L), and CD44 was detected with Alexa Fluor-488 goat anti-mouse IgG (H + L). Finally, the slides were treated with DAPI and analyzed by confocal microscopy (LSM-880, Zeiss, Germany).

### Invasion and migration assay

Cells (1 × 10^5^ cells per mL) were suspended in 0.2 mL of serum-free RPMI for the invasion and motility assays. For the invasion assay, the cells were loaded in the upper well of a transwell chamber (8 µm pore size; SPL, Korea) that had been pre-coated with matrigel (Corning, USA). The lower well was filled with 0.75 mL of RPMI supplemented with 20% serum. After incubation for 48 h at 37 °C, non-invading cells on the upper surface of the filter were removed with a cotton swab. Invading cells on the lower surface of the filter were fixed and stained with a crystal violet menthol solution (Sigma-Aldrich, USA), and then photographed at ×20 magnification. Invasiveness was determined by counting cells in four microscopic fields per well. The extent of invasion is expressed as the average number of cells per microscopic field.

For migration studies, we used invasion chambers with control inserts that contained matrigel. Cells (5 × 10^4^ cells per mL) in 0.2 mL of serum-free RPMI were added to the apical side of each insert, and 0.75 mL of RPMI supplemented with 20% serum was added to the basal side. The inserts were processed as described above.

### Western blot

Cell extracts were obtained using 1× extraction buffer (Abcam, UK) containing extraction enhancer buffer (Abcam, UK). The tumor tissue from the mice was first cut into small pieces using scissors. The tissue was then lysed by homogenization in 1× protein extraction cell lysis buffer (Abcam, UK) containing extraction enhancer buffer (Abcam, UK). Final protein concentrations were determined by BCA assay (ThermoFisher Scientific, USA). SDS polyacrylamide gels (10%) were loaded with 100 μg total protein per lane. Pre-stained protein molecular weight markers (ThermoFisher Scientific, USA) were run as standards. The electrophoresed samples were transferred to PVDF membranes. After transferring using the iBlot2 dry blotting system (Thermo Fisher Scientific), the membrane was blocked with 5% blotting-grade blocker in 1x TBST (ThermoFisher Scientific, USA) for 1 h at room temperature (RT). The membrane was then incubated with diluted primary antibodies (see **Table S6**) overnight at 4°C, rinsed and washed 3 times, and incubated with the appropriate HRP-conjugated secondary antibody (**Table S6**) for 2 h at RT. The membrane was washed and rinsed as previously described, the expressed proteins were detected using the pierce ECL western blotting substrate (Thermo Fisher Scientific), and membranes were visualized using the Amersham Imager 600 documentation system (GE Healthcare Life Sciences, Pittsburgh, PA). Each experiment was performed in triplicate.

### Immunofluorescence (IF)

After euthanization by CO_2_, tumor specimens were extracted from the mice. These were fixed with 4% paraformaldehyde in PBS overnight at 4°C. The samples were then embedded in OCT compound and frozen at -20 °C. Transverse 8 μm-thick tumor sections were cut and then permeabilized using 0.2% Triton-X solution. The sections were blocked using 10% normal goat serum for 1 h at room temperature, and then processed with CD44, E-cadherin, N-cadherin, and PD-L1 antibodies overnight at 4 °C. After the sections were processed with the secondary antibody (see **Table S6**), they were mounted using DAPI mounting solution. Images were then acquired using confocal microscopy (LSM-880, Zeiss, Germany).

### Quantitative real-time polymerase chain reaction (qPCR)

Total RNA was extracted from cells using TRIzol (Molecular Research Center, USA) in accordance with the manufacturer's instructions. The tumor tissue from the mice was cut into small pieces using scissors. The tissue was then lysed by homogenization in TRIzol. Total RNA was subsequently extracted from the tissue lysis sample. Isolated RNA was quantified by spectrophotometry, and then diluted in RNase-free dH_2_O (ThermoFisher Scientific, USA). For the PCR, a SuperScript III cDNA synthesis Kit (Invitrogen, USA) was used to generate cDNA from 5 µg of mRNA following the manufacturer's instructions. qPCR was performed on an ABI qPCR system (Applied Biosystems, USA) using SYBR Green PCR Master Mix (Applied Biosystems, USA) following the manufacturer's instructions. The reaction conditions were: 10 min at 95 °C; 50 cycles of 95 °C for 15 s and 60 °C for 60 s. The primer sequences are shown in **Table S6**. mRNA values were normalized to that for GAPDH. Relative quantity (RQ) values for mRNA expression were calculated using the equation RQ = 2^-ΔΔCt^.

### Knockdown of ASGR2 expression using siRNAs

ASGR2 knockdown was in accordance with the manufacturer's instructions. ASGR2-specific siRNA sequences were designed using a web-based tool (Bioneer, Rep. of Korea and **Table S6**). ASGR2-specific or control, nonspecific siRNAs (40 nmol/L of each; siControl; Bioneer, Rep. of Korea) were used to transiently transfect AGS, MKN1, MKN45, NCI-N87, and KATOIII cells in the presence of Lipofectamine 3000 (Invitrogen, USA) 3 days after splitting the cell cultures.

### TCGA-STAD and the Human Protein ATLAS

Overall survival (OS) analysis data was performed using the human protein ATLAS web toolkit (https://www.proteinatlas.org/) 67. RNA-seq data from 354 patients in the cancer genome atlas-stomach adenocarcinoma (TCGA-STAD) dataset was analyzed. The demographic data is listed in **Table S5**.

Based on the FPKM value of each gene, patients were classified into two expression groups (high and low), and the correlation between expression level and patient survival was examined. To classify the two expression categories, the best expression cut-off value in the protein ATLAS web toolkit was used. The prognosis of each group of patients was examined by Kaplan-Meier survival estimators, and the survival outcome of the two groups were compared by log rank test. The overall survival (OS) results included all Stage I-IV and N/A patients. Significant OS was determined using FPKM values above 1 (as recommended by the human protein ATLAS) and a *P* < 0.0001.

### RNA isolation and RNA sequencing

To confirm the changes in gene expression in gastric tissues due to PS exposure, mice were orally administered 1.72 × 10^4^ particles /mL of PS microspheres of size 10 μm for 4 weeks. Total RNA was extracted from the stomach tissues. Three sets of RNA were collected per group. Briefly, the RNA was randomly fragmented for sequencing with short reads, and then reverse-transcribed to cDNA using distinct adapters at the 5′ and 3′ ends. PCR amplification was performed (optimized so that sequencing was still possible), and size selection was performed to obtain an insert size of 200-400 bp. These were then sequenced using the NovaSeq 6000 Sequencing System (Illumina, USA) according to the manufacturer's instructions.

### RNA-seq data analysis

RAW seq data was converted to fastq format using bcl2fastq. The raw reads from the sequencer were preprocessed to remove low quality and adapter sequences before analysis using STAR 68. The processed reads were then aligned to the *Mus musculus* genome (mm10) using HISAT v2.1 69. HISAT utilizes two types of indexes for alignment (a global whole-genome index, and tens of thousands of small local indexes). These two types of indexes are constructed using the same BWT (Burrows-Wheeler transform)/graph FM index (GFM) as bowtie2 70. By using these efficient data structures and algorithms, HISAT generates spliced alignments several times faster than the more widely used Bowtie and BWA. The reference genome sequence of *Mus musculus* (mm10) and annotation data was downloaded from the NCBI. Transcript assembly (known transcripts, novel transcripts, and alternative splicing transcripts) are processed by StringTie v1.3.4d 71, 72. The expression abundance of both transcript and gene were then calculated as read count or FPKM value per sample. The resulting expression profiles were used in additional analyses, including DEG (differentially expressed gene) analysis 73. In groups with different conditions, DEGs or transcripts were filtered through statistical hypothesis testing.

For variant calling of RNA-seq data, trimmed reads were aligned to *Mus musculus* (mm10) using STAR 68. The aligned reads were used for variant calling with the mpileup module of Samtools 74 and the call module of BCFtools 75. Variant filtering for each sample was performed based on root mean square mapping quality (Q ≥ 20) and read depth (d ≥ 100) using the varFilter module of vcftools 76.

### Statistical analysis of gene expression level

The relative abundance of genes were measured in read count using StringTie v1.3.4d 71, 72. The statistical significance of the differential expression data was determined using DESeq2 nbinomWaldTest 73 and fold change (the null hypothesis was that no difference exists among groups). We performed statistical analyses to find DEGs using the estimates of abundances for each gene in the samples 73. Genes with one or more zeroed read count values in the samples was excluded. Filtered data were log_2_-transformed and subjected to relative log expression (RLE) normalization. The false discovery rate was controlled by adjusting the *P*-value using the Benjamini-Hochberg algorithm. For the DEG set, hierarchical clustering analysis was performed using complete linkage and Euclidean distance as a measure of similarity.

### Hierarchical clustering

Hierarchical clustering analysis was performed using complete linkage and Euclidean distance as a measure of similarity to display the expression patterns of differentially expressed transcripts by using MATLAB (|fold change| > 2; raw *P* < 0.05).

### Statistical analyses

The data is presented as mean with SD. Statistical analyses were performed using PRISM (ver. 5.0. GraphPad, San Diego, CA). A two-tailed Student's t-test was used. * denotes P < 0.05; ** denotes P < 0.005, n.s denotes not significant.

## Supplementary Material

Supplementary figures.

Supplementary tables.

## Abbreviations

MP: Microplastic
DOTA: 1,4,7,10-Tetraazacyclododecane-1,4,7,10-tetraacetic acid
PS: polystyrene
PE: polyethylene
DAPI: 4′,6-diamidino-2-phenylindole
TBST: tris-buffered saline with 0.1% Tween® 20 detergent
HRP: horseradish peroxidase
PBS: Phosphate-Buffered Saline
FBS: fetal bovine serum
ECL: enhanced chemiluminescence
cDNA: complementary DeoxyriboNucleic Acid
BSA: Bovine Serum Albumin
GAPDH: glyceraldehyde 3-phosphate dehydrogenase
ASGR2: asialoglycoprotein receptor 2
PD-L1: Programmed cell death 1 ligand 1
PITX2: pituitary homeobox 2
EQTN: Equatorin
PLEKHS1: pleckstrin homology domain-containing family S member 1 (PLEKHS1)
TZB: Trastuzumab
HER2: Human epidermal growth factor receptor 2
EGFR: Epidermal growth factor receptor
MAPK: mitogen-activated protein kinase
RIT: radioimmunotherapy
mAb: monoclonal antibody
MDR: Multidrug resistance
EMT: Epithelial mesenchymal transition
FPKMs: Fragments Per Kilobase of transcript per Million mapped reads
RNA: Ribonucleic acid
mRNA: messenger Ribonucleic acid
IF: Immunofluorescence
qPCR: Quantitative polymerase chain reaction
ICC: immunocytochemistry
DEG: differentially expressed genes
micro-ATR-FTIR: micro attenuated total reflection Fourier transform infrared spectroscopy
PET: Positron emission tomography
