# Supplementary material for: Enhanced ASGR2 by microplastic exposure leads to resistance to therapy in gastric cancer
Source: Theranostics. 2022 Apr 4;12(7):3217–36. doi: 10.7150/thno.73226 (PMC9065185; doi:10.7150/thno.73226)
Supplement: Supplementary file 1 — Supplementary figures. [file thnov12p3217s1.pdf]

Supplementary Materials

Supplementary Materials for

Enhanced ASGR2 by microplastic exposure leads to resistance to therapy in gastric cancer

Hyeongi Kim[1,2], Javeria Zaheer[1,3], Eui-Ju Choi[2], and Jin Su Kim[1,3,*]

Correspondence to: kjs@kirams.re.kr

This PDF file includes:

Figs. S1 to S11

Tables S1 to S6

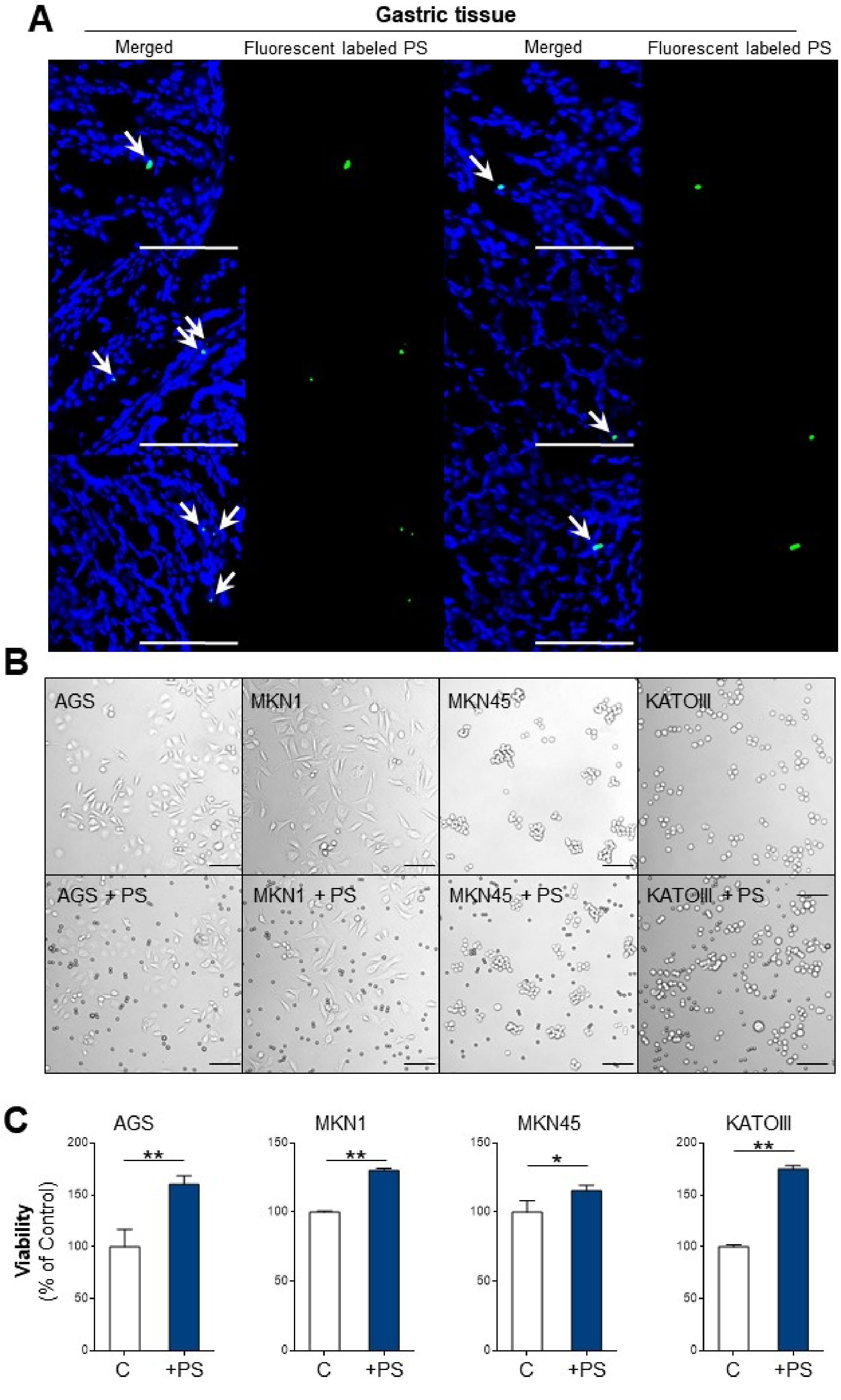
A
Gastric tissue
Merged
Fluorescent labeled PS
Merged
Fluorescent labeled PS
B
AGS
MKN1
MKN45
KATOIII
AGS + PS
MKN1 + PS
MKN45 + PS
KATOIII + PS
C
AGS
MKN1
MKN45
KATOIII
Viability
(% of Control)
C
+PS

Fig. S1. Polystyrene (PS) exposure promotes cell proliferation.

(A) Representative microscopy images showing accumulation of fluorescent green-labeled microplastic (MP) polystyrene (PS) in gastric tissues (magnification, 20×; scale bar, 100 μm). BALB/c nude mice were exposed to fluorescent green PS (1.72×10^4 particles /mL) daily for 4 weeks. The accumulation of fluorescent green-PS was identified via confocal microscopy of the harvested gastric tissues. We found that fluorescent green-PS was deposited in gastric tissues (magnification, 20×; scale bar, 100 μm).

(B-C) Microscopy images of AGS, MKN1, MKN45, and KATOIII cells with/without PS exposure and proliferation. PS exposure induced increased proliferation in AGS, MKN1, MKN45, and KATOIII cells compared with the control (mean ± standard deviation [SD], * $P < 0.05$, magnification, 20×; scale bar, 100 μm).

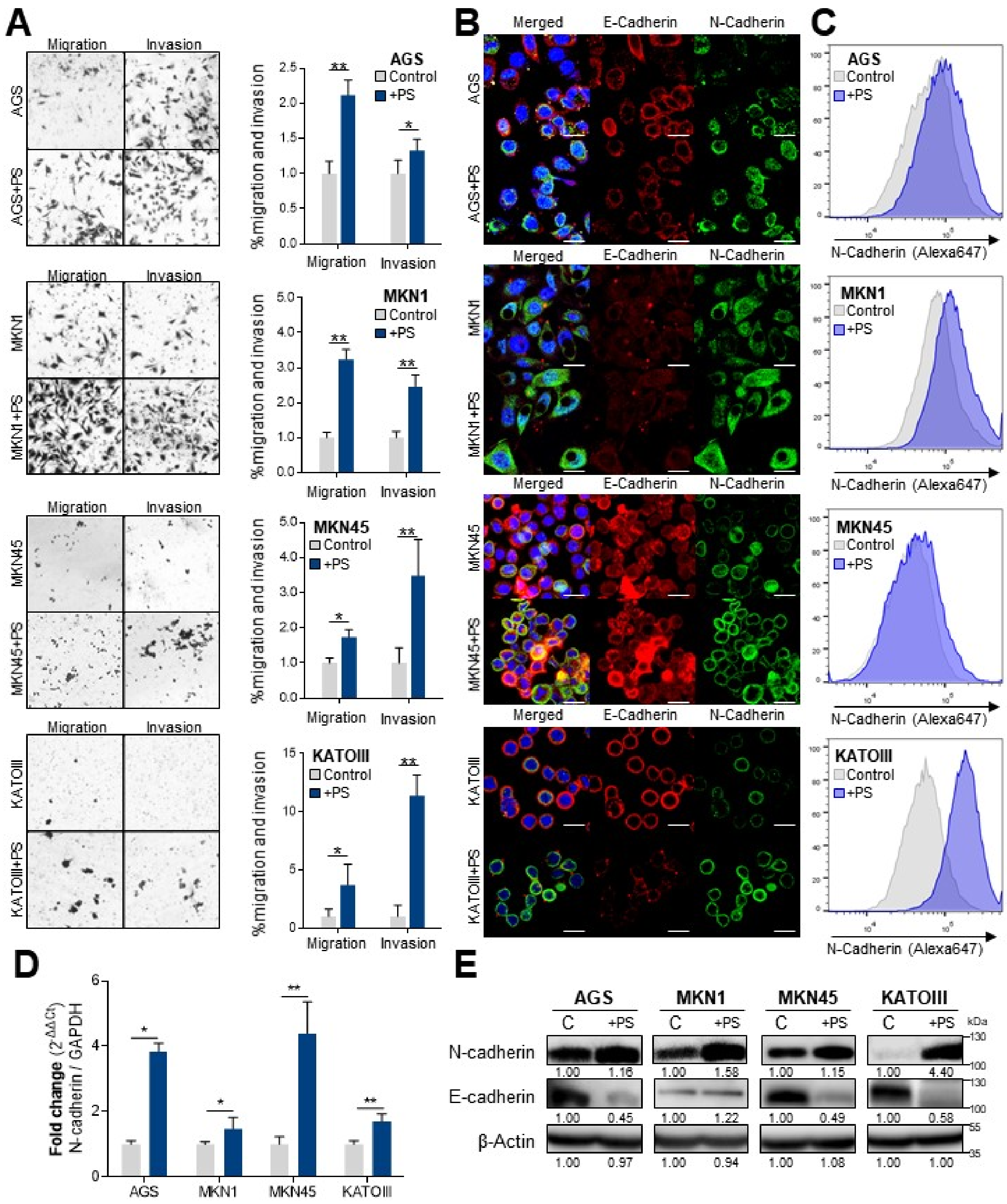

Fig. S2. PS exposure increased N-cadherin and decreased E-cadherin expression.

(A) *In vitro* migration and invasion assays. Bar graphs represent the average number of cells on the underside of the membrane, normalized to the control condition. PS promoted invasion and migration in every gastric cancer cell line (Magnification, 20×; mean ± standard deviation [SD],

*$P < 0.05$, ** $P < 0.005$, n.s., not significant. Student's t-test).

(B) Immunocytochemistry images showing gastric cancer cells stained for E-cadherin and N-cadherin. PS exposure (10 μm diameter, 8.61×10^5 PS particles/mL, 4 weeks) decreased E-cadherin levels in AGS and KATOIII cells and increased N-cadherin level in AGS and KATOIII cells (magnification, 40×; scale bar, 20 μm).

(C) Flow cytometry histograms of N-cadherin expression in gastric cancer cell lines with PS exposure. PS exposure upregulated N-cadherin expression in gastric cancer cells.

(D) Quantitative polymerase chain reaction (qPCR) analysis of N-cadherin mRNA expression. mRNA expression of N-cadherin increased after PS exposure (*$P < 0.05$, ** $P < 0.005$).

(E) Western blot analysis of N-cadherin and E-cadherin expressions with PS exposure.

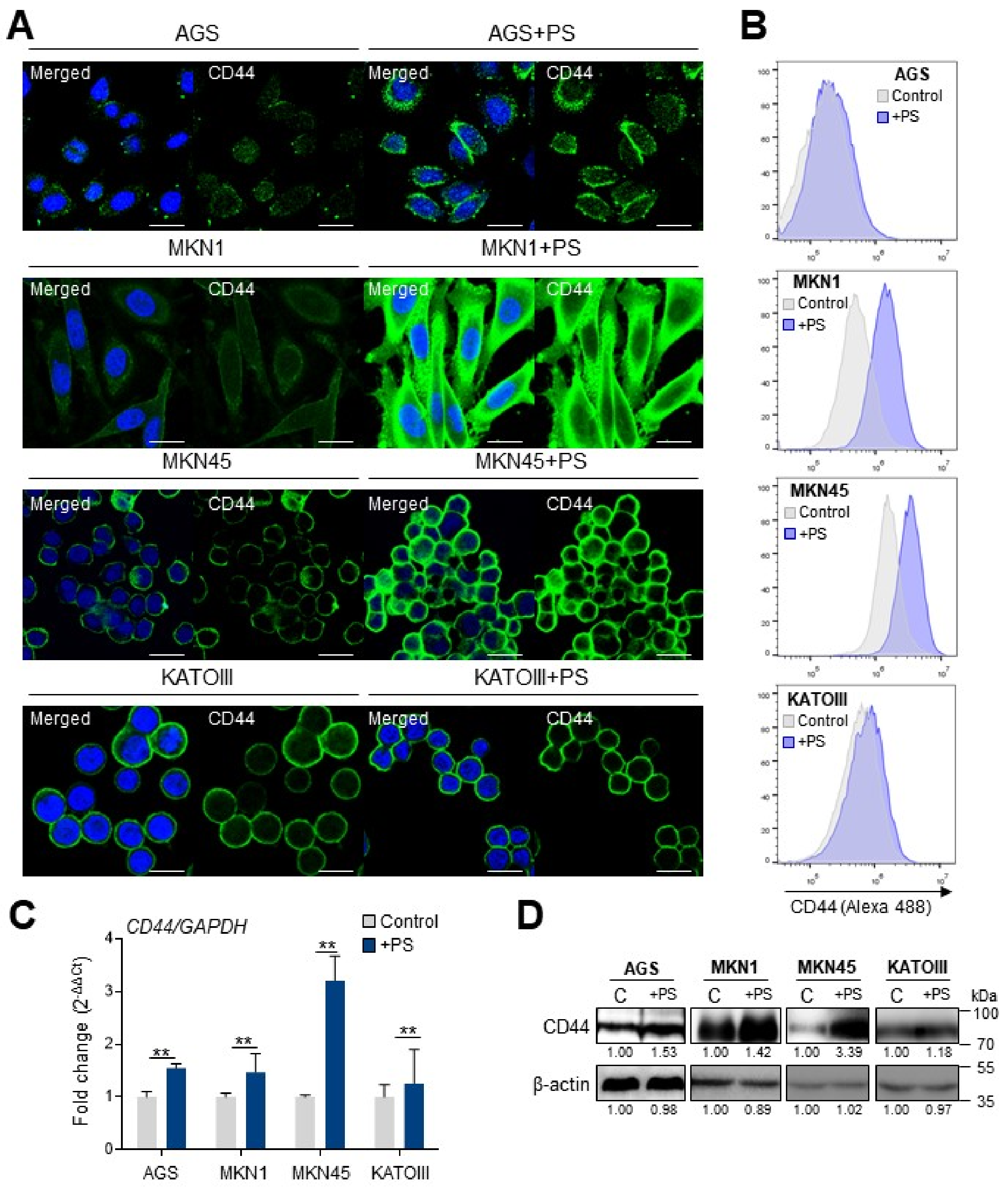

Fig. S3. PS exposure promoted upregulation of CD44 expression.

(A) Immunocytochemistry staining showing CD44 expression in gastric cancer cells (magnification, 40×; scale bar, 20 μm).

(B) Flow cytometry analysis of CD44 expression in gastric cancer cell lines. PS exposure for 4 weeks increased CD44 expression.

(C) Quantitative polymerase chain reaction (qPCR) analysis of CD44 mRNA expression (*P <

0.05, $^{**}P < 0.005$, n.s., not significant).

(D) Western blot analysis of CD44 expression in cells.

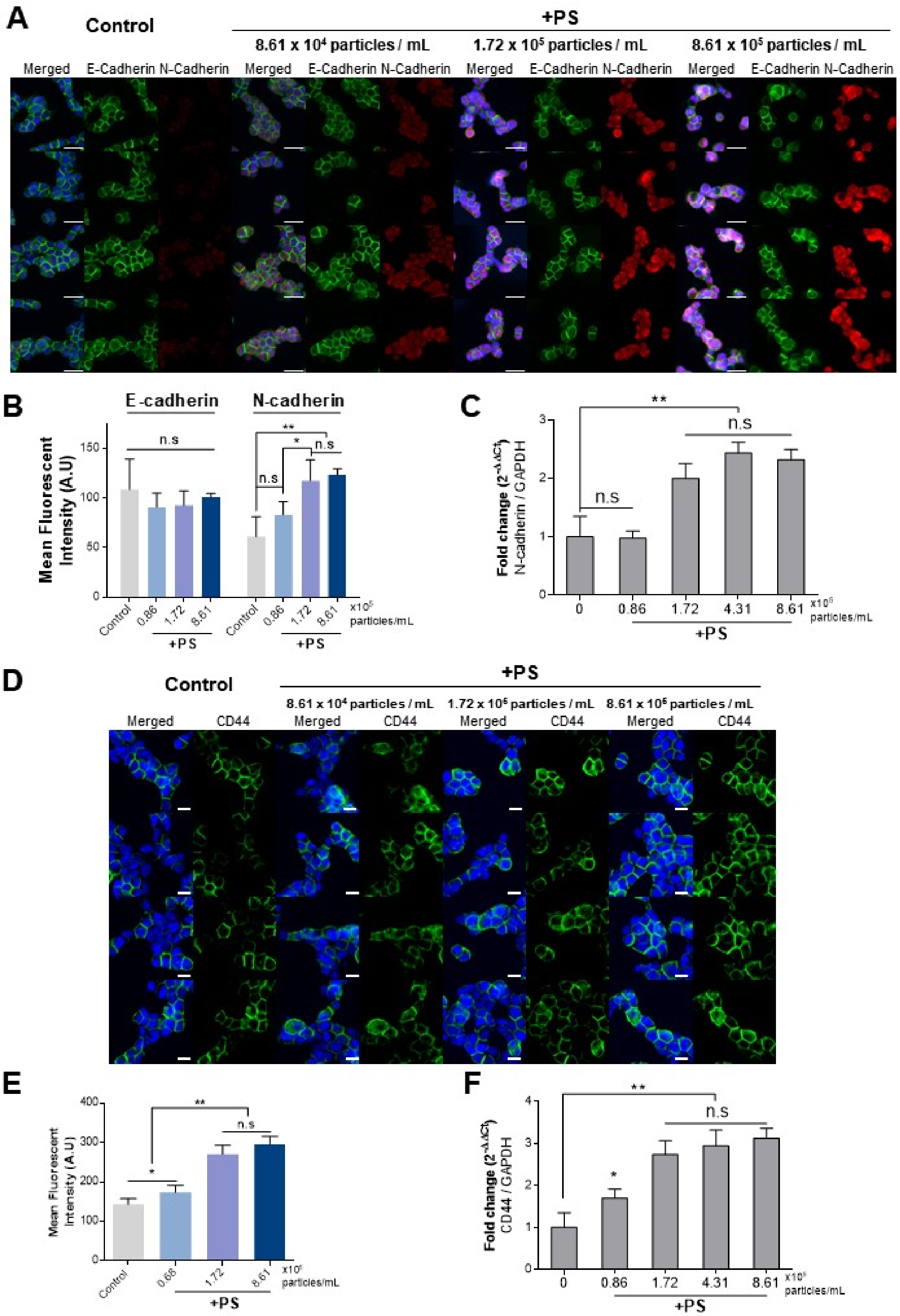
A
Control
+PS
8.61 x 10⁴ particles / mL
1.72 x 10⁵ particles / mL
8.61 x 10⁵ particles / mL
Merged
E-Cadherin
N-Cadherin
B
E-cadherin
N-cadherin
Mean Fluorescent Intensity (A.U)
n.s
**
*
Control
0.86
1.72
8.61
x10⁵ particles/mL
+PS
C
Fold change (2^-ΔΔCt) N-cadherin / GAPDH
0
0.86
1.72
4.31
8.61
D
CD44
E
F
Fold change (2^-ΔΔCt) CD44 / GAPDH

Fig. S4. PS exposure increased N-cadherin and CD44 expression in a PS concentration-dependent manner.

(A) Immunocytochemistry images showing gastric cancer cells stained for E-cadherin and N-cadherin. PS exposure (10 μm diameter, 8.6×10^4 to 8.61×10^5 particles/mL, daily for 4 weeks) increased N-cadherin expression in NCI-N87 (magnification, 20×; scale bar, 20 μm).

(B) The analysis of mean fluorescent intensity (MFI) of E-cadherin and N-cadherin. (mean ± SD, n.s; not significant, **P < 0.005, Student's *t*-test).

(C) qPCR analysis of N-cadherin mRNA expression in PS-exposed (mean ± SEM, n.s; not significant, *P < 0.05, **P < 0.005, Student's *t*-test).

(D) Immunocytochemistry images showing gastric cancer cells stained for CD44. PS exposure (10 μm diameter, 8.6×10^4 to 8.61×10^5 particles/mL, daily for 4 weeks) increased CD44 expression in NCI-N87 (magnification, 20×; scale bar, 20 μm).

(E) The analysis of mean fluorescent intensity (MFI) of CD44. (mean ± SD, n.s; not significant, **P < 0.005, Student's *t*-test).

(F) qPCR analysis of CD44 mRNA expression in PS-exposed cells (mean ± SEM, n.s; not significant, *P < 0.05, **P < 0.005, Student's *t*-test).

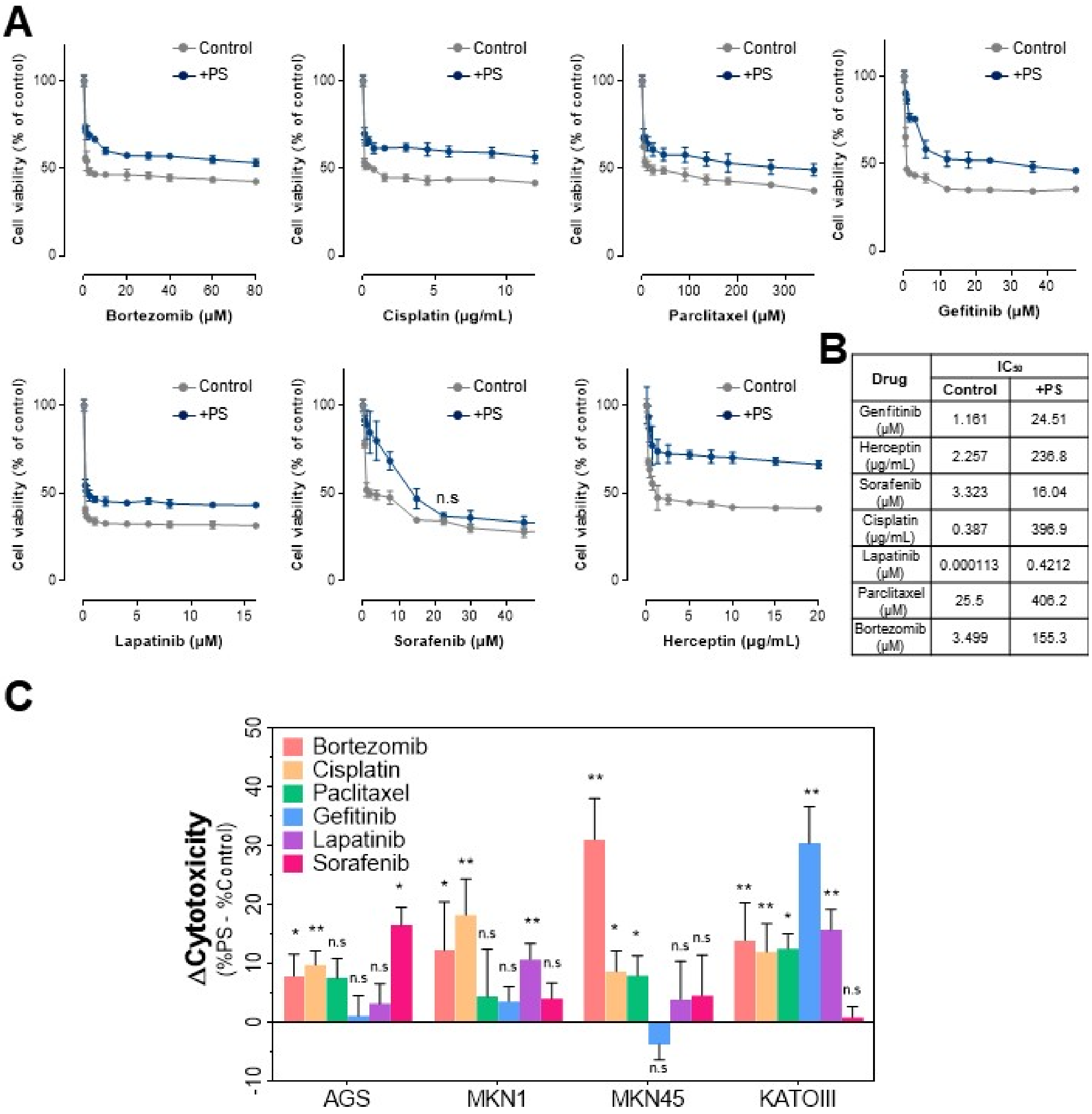

Drug	IC_{50}	
	Control	+PS
Genfitinib (μM)	1.161	24.51
Herceptin (μg/mL)	2.257	236.8
Sorafenib (μM)	3.323	16.04
Cisplatin (μg/mL)	0.387	396.9
Lapatinib (μM)	0.000113	0.4212
Parclitaxel (μM)	25.5	406.2
Bortezomib (μM)	3.499	155.3

Fig. S5. PS exposure promoted drug resistance in gastric cancer cell line.

(A) Dose-response curve to chemotherapy drugs upon PS exposure in NCI-N87 cells. (10 μm diameter, 8.61×10^5 particles/mL, daily for 4 weeks). All data presented are significant, determined using a Student's *t*-test, except those marked with "n.s."

(B) IC_{50} values. PS exposure considerably increased the IC_{50} values in NCI-N87 cells.

(C) CD44-induced drug resistance following PS exposure (10 μm diameter, 8.61×10^5 particles/mL, daily for 4 weeks) in AGS, MKN1, MKN45, and KATOIII cells. The cytotoxicity of bortezomib, cisplatin, paclitaxel, gefitinib, lapatinib, and sorafenib was measured as follows: Δcytotoxicity = cytotoxicity with PS - cytotoxicity without PS.

Each value is represented as a percentage of drug vehicle control (dimethyl sulfoxide [DMSO] or phosphate-buffered saline [PBS]). (* $P < 0.05$, ** $P < 0.005$).

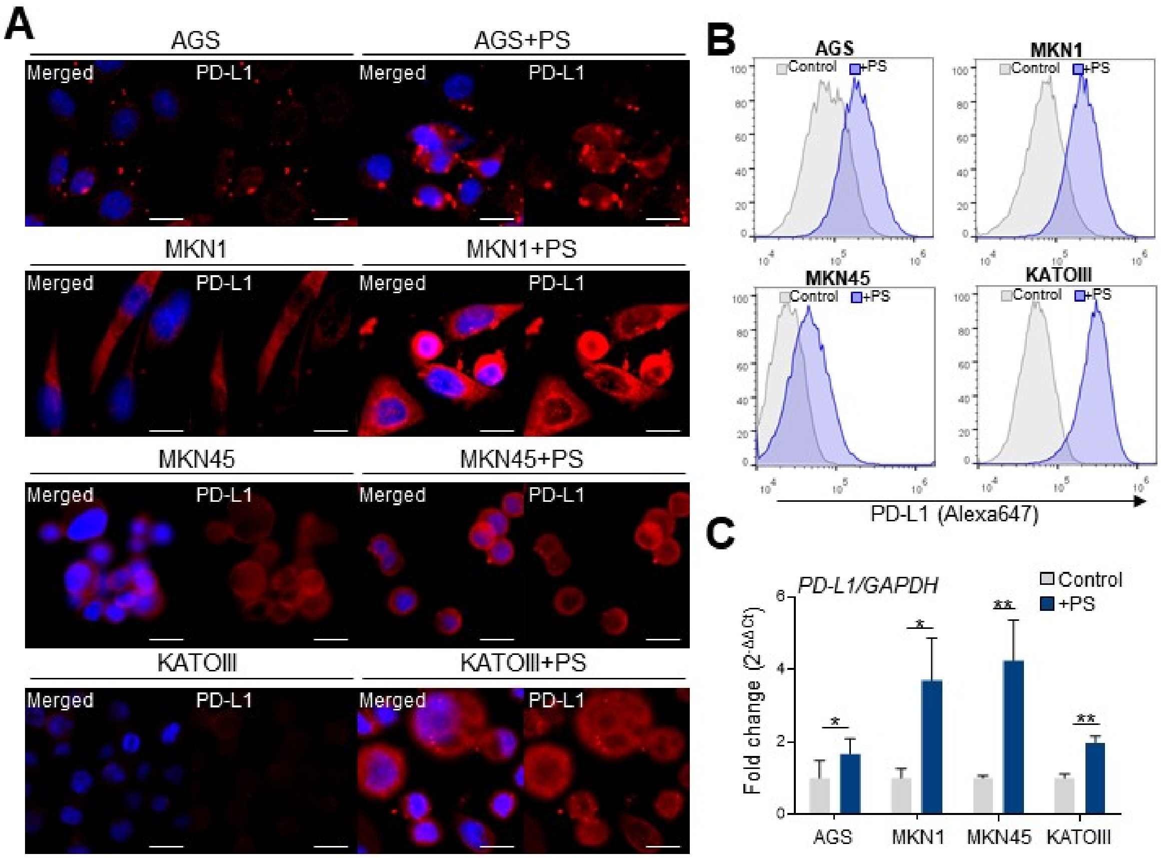

Fig. S6. Polystyrene (PS) exposure promoted upregulation of PD-L1 expression.

(A) Immunocytochemistry staining of PD-L1 in gastric cancer cells with/without PS (Magnification, 40×; Scale bar, 20 μm).

(B) Flow cytometry histograms of PD-L1 expression in gastric cancer cell lines. PS exposure for 4 weeks dramatically increased PD-L1 expression.

(C) qPCR analysis of PD-L1 mRNA expression. (*$P < 0.05$, **$P < 0.005$).

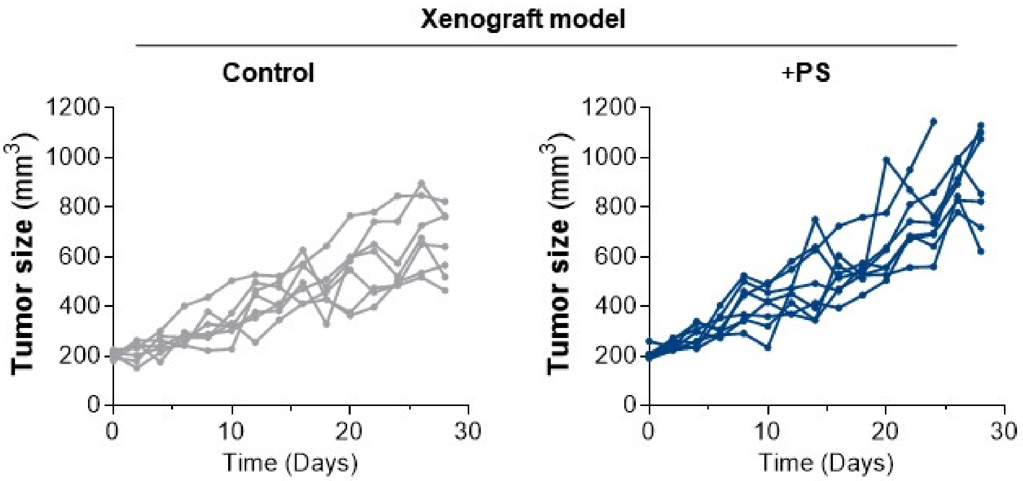

Fig. S7. PS accelerated tumor growth.
Individual tumor size for PS-exposed NCI-N87 xenograft mouse models.

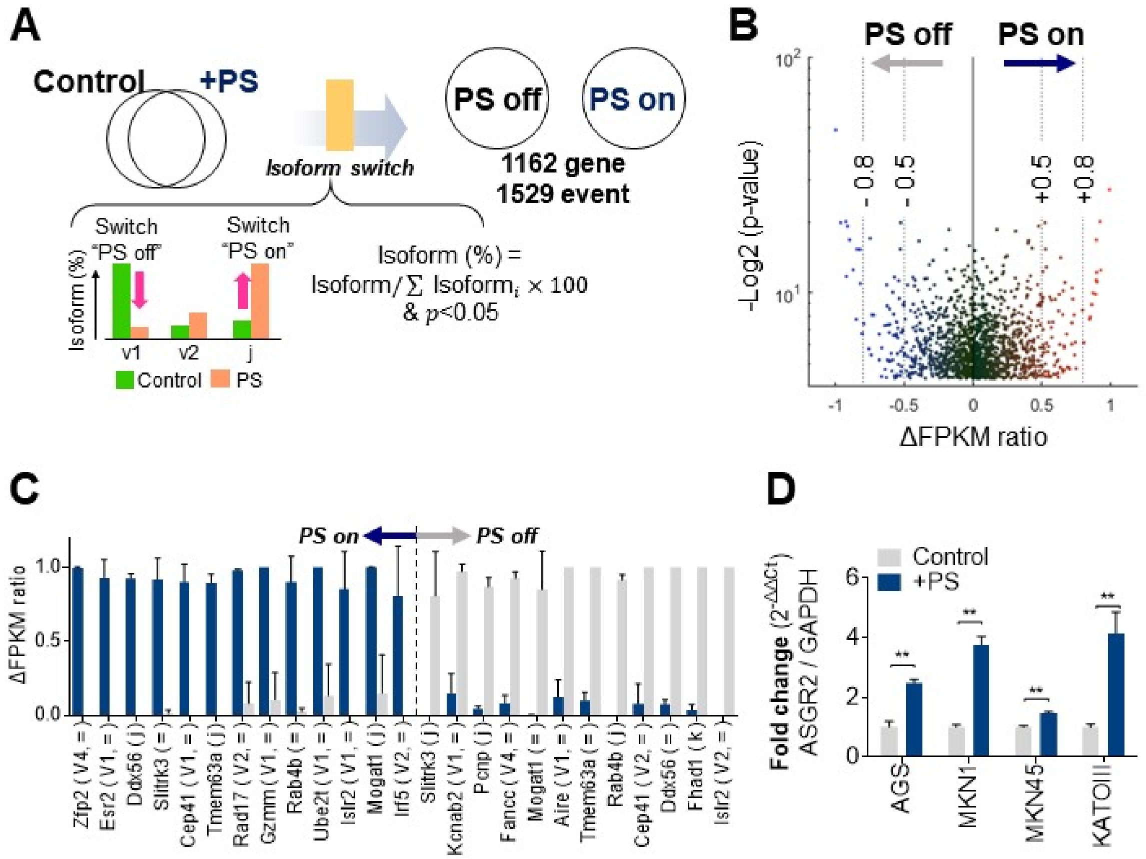

Fig. S8. PS promoted gene expression change in gastric tissue.

(A) Schematic of isoform switch analysis. The ratio of the sum of FPKMs of gene isoforms (for genes with isoforms) was calculated using the switch method (* ***P* < 0.05**). Finally, 1162 genes with 1529 events were identified (see also **Table S3**).

(B) The distribution of ΔFR change for individual gene

(C) Representative genes showing changes in ΔFR > 0.8 and ΔFR < −0.8

(D) qPCR analysis of ASGR2 mRNA expression in PS-exposed AGS, MKN1, MKN45, and KATOIII. (**P < 0.005)

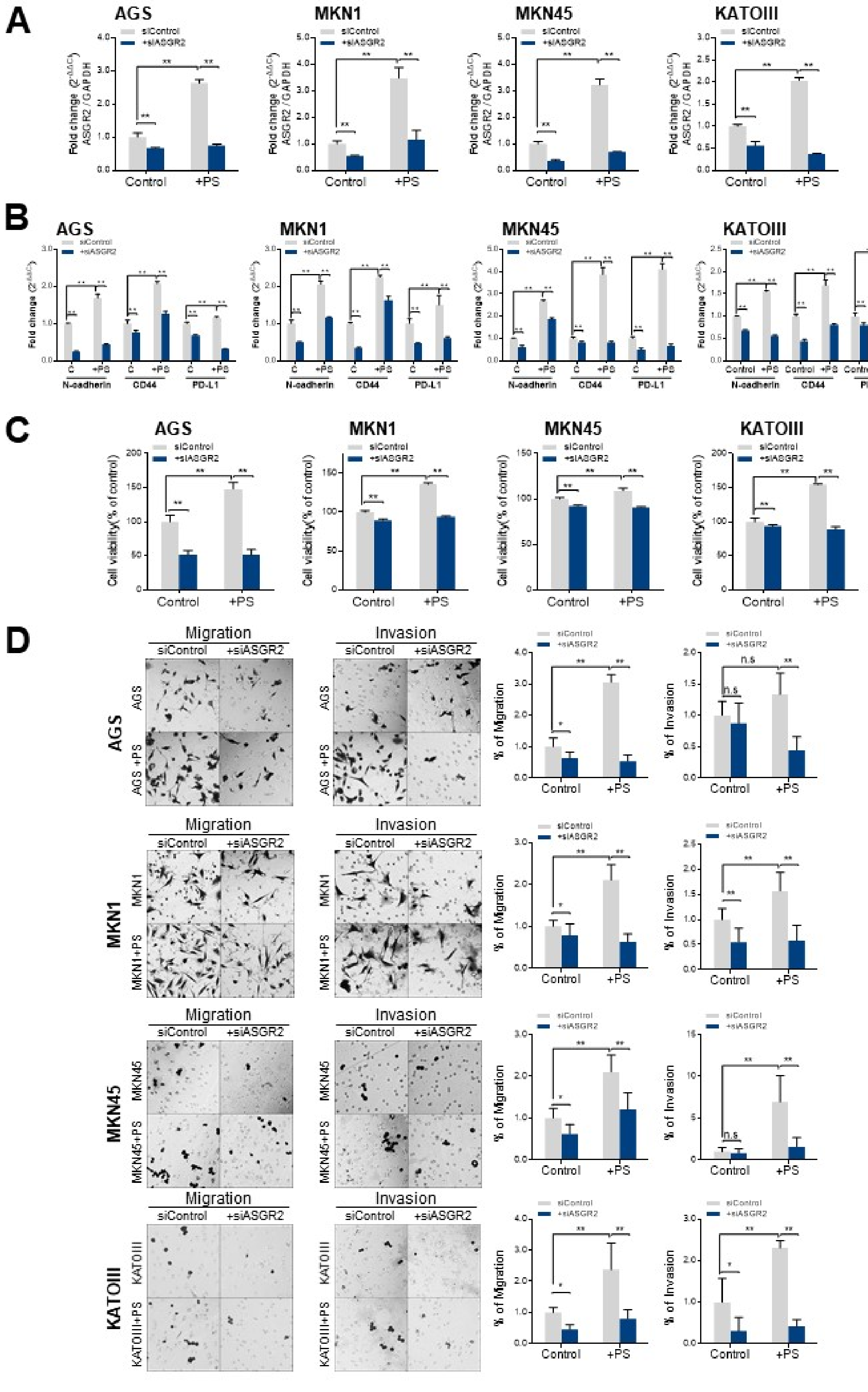
A
AGS
MKN1
MKN45
KATOIII
siControl
+siASGR2
Fold change ($2^{-\Delta\Delta C_t}$) ASGR2 / GAPDH
Control
+PS
B
N-cadherin
CD44
PD-L1
Fold change ($2^{-\Delta\Delta C_t}$)
C
Cell viability(% of control)
D
Migration
Invasion
% of Migration
% of Invasion
n.s
AGS +PS
MKN1+PS
MKN45+PS
KATOIII+PS

Fig. S9. Knockdown of ASGR2 by siRNA in AGS, MKN1, MKN45, and KATOIII

(A) Knockdown of ASGR2 by siRNA in AGS, MKN1, MKN45, and KATOIII (* $P < 0.05$, ** $P < 0.005$).

(B) qPCR analysis of N-cadherin, CD44, and PD-L1 mRNA expression in PS-exposed cell-line with knockdown of ASGR (*$P < 0.05$, **$P < 0.005$).

(C) Knockdown of ASGR2 in AGS, MKN1, MKN45, and KATOIII proliferation.

(D) In vitro migration and invasion assays. Bar graphs represent the average number of cells on the underside of the membrane, normalized to the control condition. siASGR suppressed the migration and Invasion of AGS, MKN1, MKN45, and KATOIII cells (magnification, 20×; * $P < 0.05$, ** $P < 0.005$, n.s., not significant).

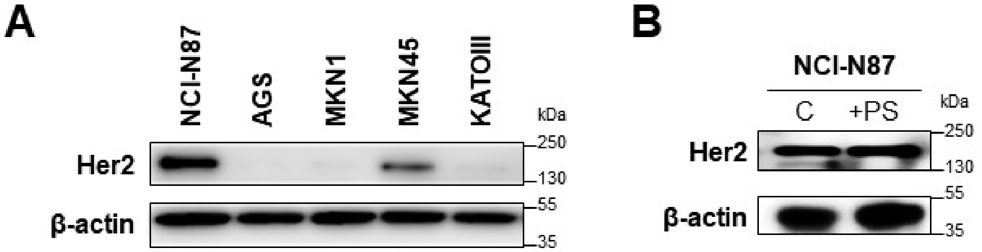

Fig. S10. Western blotting of Her2 expression in gastric cancer cells.

(A) The expression of Her2 in gastric cancer cell-line.

(B) Western blotting of Her2 expression after exposure to PS (10 μm diameter, 8.61×10^5 particles/mL, daily for 4 weeks).

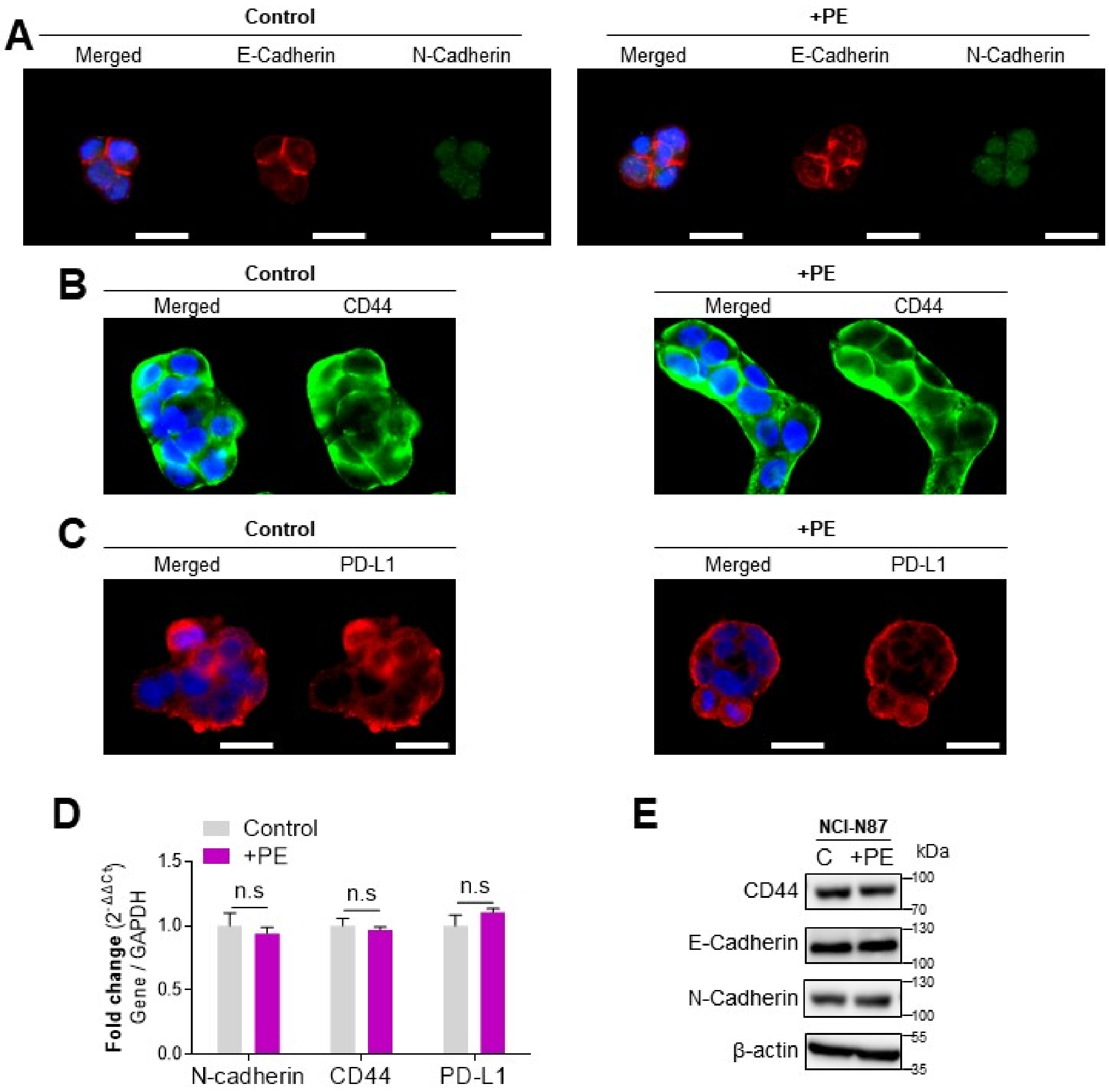

Fig. S11. Polyethylene (PE) exposure did not affect cancer hallmarks.

(A-C) Immunocytochemistry images showing NCI-N87 cells stained for E-cadherin, N-cadherin, CD44, and PD-L1. PE exposure (10-20 μm diameter, 8.61×10^5 particles / mL, 4 weeks) did not change (A) E/N-cadherin, (B) CD44, and (C) PD-L1 expression in NCI-N87 cells (Magnification, 40×; scale bar, 20 μm).

(D) qPCR analysis of N-cadherin, CD44, and PD-L1 expression (n.s; not significant).

(E) Western blotting of N-cadherin, E-cadherin, and CD44 expression with/without PE. N-cadherin, CD44, and PD-L1 did not change after PE exposure.

Supplementary Tables

Table S1. The list of DEG analysis in gastric tissue by PS exposure.

Table S2. The number of isoform changes in the gastric tissue following polystyrene (PS) exposure**.**

Table S3. List of isoform-changed genes and ΔFR.

Table S4. Isoform changes identified using the switching method

Table S5. Demographic Cancer Genome Atlas Stomach Adenocarcinoma (TCGA-STAD) datasets.

Table S6. Key resource table.
