# Supplementary material for: Enhanced ASGR2 by microplastic exposure leads to resistance to therapy in gastric cancer
Source: Theranostics. 2022 Apr 4;12(7):3217–36. doi: 10.7150/thno.73226 (PMC9065185; doi:10.7150/thno.73226)
Supplement: Supplementary file 2 — Supplementary tables. [file thnov12p3217s2.zip › Table S5.docx]

**Table S5. Demographic Cancer Genome Atlas Stomach Adenocarcinoma (TCGA-STAD) datasets**

| Variables | Counts (n=354) | |
| --- | --- | --- |
| Age(years) |  | |
| ≤60 | 241 | 68.1% |
| >60 | 107 | 30.2% |
| N/A | 6 | 1.7% |
| Gender |  |  |
| Female | 125 | 35.3% |
| Male | 229 | 64.7% |
| Pathologic stage |  |  |
| Stage I | 48 | 13.6% |
| Stage II | 110 | 31.1% |
| Stage III | 146 | 41.2% |
| Stage IV | 35 | 9.9% |
| N/A | 15 | 4.2% |

N/A: not available.
